# Probiotic properties of *Bacillus subtilis* isolates from neonatal meconium

**DOI:** 10.3389/fmicb.2026.1804128

**Published:** 2026-05-14

**Authors:** Saurabh Kumar Singh, Sadhana Sathaye, Jayraj Doshi

**Affiliations:** 1 Department of Pharmaceutical Sciences and Technology, Institute of Chemical Technology, Mumbai, India; 2 Zytex Biotech Pvt. Ltd., Mumbai, Maharashtra, India

**Keywords:** antioxidant capacity, *Bacillus subtilis*, cholesterol assimilation, gastrointestinal tolerance, neonatal meconium, spore-forming probiotics

## Abstract

Neonatal meconium represents a relatively underexplored microbial source that may harbour microorganisms with probiotic potential, uniquely adapted for intestinal colonization and metabolic health. From 120 meconium samples, 167 spore-forming isolates were screened to identify strains exhibiting desirable probiotic functional and safety attributes. Three strains (ZBOE6, ZBRV8, and ZBTM2) were selected and identified as *Bacillus subtilis* through whole-genome sequencing. These strains showed non-haemolytic (α/γ types) and DNase negative phenotypes, indicating non-pathogenic profile, along with detectable bile salt hydrolase (*Bsh*) activity. The isolates exhibited tolerance to adverse stressors, including acidic conditions, bile salts, and simulated gastric and intestinal conditions. Isolates showed epithelial adhesion potential (up to 87.5% for ZBRV8) and auto-aggregation efficiencies ranging from 60 to 80%. *In vitro* assays using Caco-2 cell lines demonstrated anti-adhesive potential against *E. coli* through competitive, exclusion, and displacement mechanisms. The strains showed cholesterol assimilation potential greater than 60% and antioxidant activity up to 62.7% (DPPH scavenging) and 97.9% (hydroxyl radical scavenging). *In vitro* safety assessment revealed no biogenic amine production, minimal cytotoxicity (<30%) in the Vero cell line, and susceptibility to clinically relevant antibiotics with minor strain-dependent variation consistent with intrinsic resistance profiles. Enzyme assay profiling revealed activity for lipase, phytase, amylase, and β-galactosidase. Genomic analysis further supported these phenotypic observations by identifying genes associated with stress tolerance, adhesion, metabolism, and antimicrobial activity, providing preliminary mechanistic insight into strain-specific functional traits. Overall, these findings suggest that *Bacillus subtilis* isolated from human neonatal meconium represent promising probiotic candidates; however, their functional efficacy and safety require further validation through *in vivo* and clinical studies for gut and metabolic health interventions.

## Introduction

The human gastrointestinal tract hosts a highly dynamic ecosystem composed of a diverse range of microorganisms, including bacteria, archaea, viruses, and fungi, collectively referred to as the gut microbiota. Within this complex microbial ecosystem, probiotics have attracted considerable interest in both scientific studies and clinical application. At present, the popularity in probiotics is increasing due to the high consumer demand for safe and functional foods that offer health benefits and possess high nutritional content. Probiotics are defined as “live microorganisms that, when administered in adequate amounts, confer a health benefit on the host” (Kechagia et al., 2013). Although different microorganisms are claimed today as probiotics, they are mostly associated with *Lactobacillus* and *Bifidobacterium* (Ritter et al., 2018).

*Bacillus* species have also been investigated as probiotic candidates because of their resilience to harsh food processing conditions, such as high pressure and temperature tolerance, and better survival rates in the gastrointestinal tract. They are also known to have a better shelf life and remain viable throughout the lifetime at room temperature (RT) and under refrigeration (Payne et al., 2024).

Meconium, the first faecal discharge from neonates, is a viscous, dark green substance and represents a unique milieu for the isolation of potentially probiotic bacteria (Al Atya et al., 2015). Unlike subsequent faecal matter, meconium is composed of materials, including intestinal epithelial cells, mucus, bile, and swallowed fluid, that is ingested in utero. The composition offers a preliminary snapshot of the intrauterine environment and the intestinal microbes that may impact neonatal gut microbiome establishment (Rodríguez et al., 2015). Research carried out in Indonesia has revealed that the meconium microbiota is dominated by *Staphylococcus*, *Bacillus,* and *Enterococcus faecalis* (Oktaviyani et al., 2021). Previous studies have shown that gut microbiota of Indian and Western infants are broadly comparable with higher abundances of the *Bifidobacterium, Bacteroides,* C*lostridium, and Lactobacillus genera* (Xu et al., 2020), irrespective of whether delivery occurred vaginally or by caesarean section.

While popular probiotic candidates have been lactic acid bacteria (LAB) and bifidobacteria, spore-forming *Bacillus* species have also received considerable attention as probiotic candidates (Golnari et al., 2024). Some notable spore former species that have shown probiotic properties are *Bacillus laterosporus, Bacillus subtilis, Bacillus clausii, Bacillus licheniformis,* etc. (Lee et al., 2017; Shivangi et al., 2020). Compared with conventional probiotic, spore-forming bacteria offer notable advantage including prolonged stability at RT and enhanced tolerance to adverse gastrointestinal condition such as acidic pH, high temperature, elevated bile salt concentration, nutrient starvation, and desiccation (Golnari et al., 2024).

Although *Bacillus* sp. has been isolated and characterized from diverse sources, strains derived from human neonatal meconium have received limited systematic evaluation for probiotic potential. This study addresses this gap by isolating, identifying, and comprehensively characterizing *Bacillus subtilis* from neonatal meconium for probiotic potential.

## Materials and methods

### Preparation of samples and isolation of *Bacillus* sp.

Meconium samples were obtained from the faeces of <24-h old newborns at Ruxmani lying-in Hospital, Mumbai, India, in accordance with protocols approved by the Institutional Ethics Committee (Approval No. RLH/10/2021/P15). Samples were collected in gamma irradiated sample containers and transported to the laboratory in ice bags. The collected samples were maintained in the laboratory at 2–8 °C before further analysis. In brief, 1–10 g of meconium was mixed with 9–90 mL of TSB (tryptic soy broth). Appropriate dilutions were made in 0.85% (w/v) saline, and 0.1 mL was spread on tryptic soy agar (TSA). The plates were incubated aerobically at 37 °C for 24–48 h (Khan et al., 2023). The isolated colonies with distinct morphologies were purified followed by Gram staining. Gram-positive colonies were selected for spore staining via the Schaeffer–Fulton method (Black, 2006). Colonies showing spores were selected for subsequent characterization. The isolates were maintained at 4 °C for short-term storage, whereas reference culture was cryopreserved at −80 °C in 20% glycerol stock for long-term preservation. The presumptive *Bacillus* isolates were identified via whole-genome sequencing using an Illumina MiSeq DNA sequencer.

### Identification (whole-genome sequencing and bioinformatics assessment)

Genomic sequencing was carried out to unravel/determine the taxonomic identification and probiotic functional potential. For each strain, a single well-isolated colony from TSA plate was selected, and the genomic DNA was extracted and processed for sequencing via a Nextera XT library preparation kit (Illumina, Inc.). Sequencing was performed on an Illumina MiSeq platform using MiSeq V3 2 × 300 sequencing kit in accordance with manufacturer’s guidelines. Reads passing quality filtering at a Q20 threshold were assembled using CLC bioGenomics workbench 20.0 (Qiagen Inc., Cambridge, MA) with the default settings to generate draft genomes. Assembly quality and completeness were assessed with QUAST v5.2.0 and CheckM v1.2.2, respectively.

Functional annotations were performed using Rapid Annotation using Subsystem Technology (RAST) and Bakta v1.8.2 for comprehensive gene prediction and subsystem classification. Probiotic relevant subsystems were manually curated for stress response genes (acid/osmotic/oxidative), bile salt hydrolase (BSH), sporulation germination, biofilm formation, carbohydrate utilization, and metabolic biosynthesis. Safety screening targeted virulence factors, haemolysins, and transferable antibiotic resistance genes using CARD and VFBD database. Gene count represents subsystem assigned loci with >80% identity to reference sequence.

Taxonomic assignment was first performed by BLAST analysis of the gyrB gene against the NCBI nucleotide database followed by genome level confirmation using the Type (Strain) Genome Server (TYGS) to obtain DNA–DNA hybridization-based species identification and by calculating average nucleotide identity (ANI) values against reference genomes. The assembled draft genomes were deposited in GenBank as whole-genome shotgun submission.

#### Sporulation capacity

The spore formation efficiency of the isolates was checked in TSB supplemented with 0.2% CaCl_2_ and MnSO_4_ (Sinnelä et al., 2019). Isolates were incubated in TSB broth at 37 °C for 24–48 h, and sporulation efficiency was assessed via spore staining (Black, 2006).

#### Haemolysis assessment (*in vitro* and in silico)

Haemolytic activity was assessed by streaking the overnight grown pure culture of the isolate on ready-to-use sheep blood agar plates (HiMedia, India) via the four-flame method followed by incubation at 37 °C for 48 h. Colonies surrounded by greenish zone were considered α-haemolytic, those with clear zone as β-haemolytic, and isolates showing no visible zone were considered as γ or non-haemolytic (Lee et al., 2017). *In silico* screening of whole-genome assemblies for haemolytic genes was performed using v1.0.1 against NCBI and VFDB databases, with Bakta v1.9.3 annotation (Schwengers et al., 2021; Myintzaw et al., 2023).

#### DNase activity

This was assessed by streaking the overnight-grown cultures on DNase agar plates followed by incubating them at 37 °C for 18–24 h (Shivangi et al., 2020; Ben Taheur et al., 2016). The presence of clear zone around the colonies were considered as DNase positive, whereas no zone considered as DNase negative.

#### Biofilm formation

Biofilm formation attribute was evaluated via crystal violet staining with some modifications (Ozkan et al., 2013). The isolates were cultured in TSB broth for 18 h at 37 °C until they reached the exponential phase. After incubation, the broth was discarded and the tubes were rinsed with distilled water before adding 1% crystal violet. The tubes were left under static conditions for 30 min, after which the excess stain was removed. The presence of thick violet rings suggests that the isolates can form biofilms. Uninoculated culture tubes and freshly inoculated culture tubes were utilized as controls.

#### Acid and bile tolerance

The acid and bile tolerance assay was performed using previously reported methods with minor modifications (Khan et al., 2023). Overnight-cultured cells were centrifuged at 8,000 rpm, and the resulting cell pellets were resuspended in phosphate-buffered saline (PBS) to obtain an initial concentration of 1 ×10^8^ CFU/mL. For acid tolerance, the suspension was adjusted to pH 2.0 and incubated for 90 min at 37 °C with shaking at 150 RPM. For bile tolerance, the suspension (1 × 10^8^ CFU/mL) was inoculated into TSB supplemented with 0.3% bile (HiMedia) and incubated for 90 min at 37 °C with shaking at 150 RPM. Following incubation, surviving cells were enumerated on TSA plates in triplicates using pour plate method. The tolerance rate (%) was calculated as (CFU_T1_/CFU_T0_) *100, where CFU is a colony-forming unit, CFU_T0_ represents the initial viable count, and CFU_T1_ represents the viable count after exposure.

#### Direct plate BSH assay

Bile salt deconjugation activity was assessed qualitatively using a direct plate assay (Kumar et al., 2012). In brief, overnight cultures of the isolates were streaked onto TSA (HiMedia, India) supplemented with 0.5% (w/v) taurodeoxycholate (TDC) and 0.37 g of calcium chloride/L (Merck, India) followed by aerobic incubation at 37 °C. Plates were observed at 24, 48, and 72 h, and the appearance of precipitated acid around colonies (opaque halo) or the formation of opaque, granular white to off white colonies was considered positive reactions for the presence of *Bsh*.

#### Auto-aggregation and co-aggregation

Auto-aggregation and co-aggregation capabilities of the isolates were assessed using previously used method (Tareb et al., 2013; Jeon et al., 2017) with some modifications. Overnight grown cultures were centrifuged at 1,000 rpm for 10 min. The pellets were washed with sterile PBS (pH 7.2) and resuspended in sterile PBS to achieve an absorbance of 0.5 ± 0.2 OD (600 nm). For auto-aggregation percentage (%), the suspension was incubated under static conditions at RT, and the optical density was measured at different time intervals. The percentage auto-aggregation was determined by 1 − (OD_time_/OD_T0_) × 100, where OD_time_ represents the optical density of the mixture at different time intervals and OD_T0_ represents the optical density at time zero.

For co-aggregation, equal volumes of the cell suspension and pathogenic strains were mixed and incubated at RT under static conditions for up to 24 h. The percentage of co-aggregation was expressed as [{(Ap + Ab/2) − Amix}/(Ap + Ab)/2] × 100, where Ap and Ab represent the absorbance of the pathogen and the absorbance of the isolated strain at 0 h, respectively, and Amix represents the absorbance of the mixed culture after a specific incubation time.

#### Microbial adhesion to hydrocarbons (MATH assay)

Isolates were cultured in TSB broth for 18 h at 37 °C, and the broth was subsequently centrifuged to obtain an OD ranging from 0.5 to 1.0 (~1 × 10^8^ CFU/mL). One millilitre of the selected solvents was added with 3 mL of cell suspension and incubated for 2 h and 24 h. The OD of aqueous suspension was measured for the aqueous phase at 600 nm. Four different solvents were tested, and bacterial adherence to the solvents was calculated using the formula: (1 − A_1_/A_0_) * 100, where A_1_ represents the optical density at 2 and 24 h, and A_0_ represents the OD at time zero (Singh et al., 2016).

#### Antibiotic susceptibility

Antibiotic susceptibility was determined by disk diffusion assay according to CLSI guidelines (Clinical and Laboratory Standards Institute, 2015). The antibiotics selected in the assay were based on the recommendations of the EFSA Panel on additives and products or substrates used in animal feed (FEEDAP, 2012) for evaluating microbial strains intended for human use as live cultures or probiotic or animal feed additives. CLSI method provided standardized zone diameter measurement and correlation to minimum inhibitory concentration (MIC), while EFSA cutoffs defined safety threshold for intrinsic *Bacillus* resistance. Overnight grown cultures were uniformly spread over the surface of Mueller–Hinton Agar (MHA) using a sterile cotton swab followed by incubation for 18–24 h at 37 °C. Antibiotic strips with known concentrations (HiMedia) were then employed over the MHA plate followed by incubation for 24–48 h at 37 °C. Susceptibility was assessed by measuring the diameter of the inhibition zone in millimetre, which reflects the diffusion of the antibiotic in the agar medium (Kharwar et al., 2022). *In silico* analysis was performed using ARBicate v1.0.1 against NCBI Bacterial Antimicrobial Resistance Reference Gene database and Comprehensive Antibiotic Resistance Database (CARD) databases with thresholds of ≥90% identity and ≥60% coverage to distinguish intrinsic from acquired resistance.

#### Antimicrobial activity against pathogens

Antibacterial activity was determined via an agar well diffusion assay against different pathogens (Ritter et al., 2018; Golnari et al., 2024; Poormontaseri et al., 2017). Pathogenic test cultures included *Escherichia coli* ATCC 8739, *Staphylococcus aureus* ATCC 6538, *K. aerogenes* ATCC13048*, Salmonella enterica* ATCC 14028, *Candida albicans* ATCC 10231, and *Pseudomonas aeruginosa* ATCC 9027. Probiotic isolates and pathogens were cultured in TSB media for 18 h at 37 °C, and the culture OD was set to 1 (~1 × 10^8^ CFU/mL). Test pathogens at 1.0 OD in PBS solution were inoculated with a swab onto preincubated Mueller–Hinton Agar (MHA) plates. An 8mm diameter was created at the centre of the plate via a sterile tip, and 50 μL of the probiotic isolate and cell-free extract (CFE) were inoculated into the well. Incubation was done aerobically at 37 °C, and the readings were monitored for 24 and 48 h. For *C. albicans* ATCC 10231, the plates were incubated at 30 °C for 48 h. The zone of inhibitions (ZOIs, represented by clear halos) was measured, and the antagonistic activity was calculated. Antagonistic activity = Zone of Inhibition (mm)/Well Diameter (mm).

#### Cholesterol assimilation

The assimilation of cholesterol by *Bacillus* sp. in broth was evaluated using the *O*-phthalaldehyde method with some modifications (Rudel and Morris, 1973; Shobharani and Halami, 2016). The isolates were cultured in LB broth supplemented with 100 μg/mL water-soluble cholesterol (Sigma) and 0.3% (w/v) bile salt (HiMedia, India) followed by aerobic incubation at 37 °C for 18 h. The mixture was subsequently centrifuged (10,000 rpm at 4 °C for 15 min), and the resulting cell-free supernatant was used for residual cholesterol estimation. For analysis, 0.5 mL of CFS was mixed with 2 mL of KOH (33% w/v) and 3 mL of absolute ethanol (Hayman), vortexed for 1 min, and incubated at 37 °C for 15 min. After cooling, 2 mL of distilled water and 5 mL of hexane (Sigma) were added, and the mixture was vortexed for 1 min and kept idle for phase separation. A total of 2.5 mL of the hexane layer were transferred into a glass tube and evaporated via a water bath set at 80 °C. After evaporation, the residue was immediately dissolved in 4 mL of *O*-phthalaldehyde reagent (HiMedia, India) (0.5 mg/mL acetic acid), vortexed for 1 min, and left at RT for 10 min. Two millilitres of concentrated sulphuric acid (SD FCL) was added slowly to the mixture and vortexed for 1 min. The mixture was left idle at RT for 10 min, and the absorbance was measured at 550 nm via a UV Vis spectrophotometer (Shimadzu UV-1900i). The percentage reduction in cholesterol level was determined by the difference between the cholesterol level in the control (uninoculated LB broth) and test samples (CFS of culture).

#### *In vitro* cytotoxicity studies via the MTT assay

The cytotoxicity was evaluated using the Vero cell line as described previously with some modifications (Poormontaseri et al., 2017; Rohith and Halami, 2021). In brief, 1 × 10^4^ cells per well were seeded (minimum essential medium-MEM with 10% faecal bovine serum—FBS) in a 96-well plate (in triplicates) followed by incubation at 37 °C for 24 h at 5% CO_2_. The cells were allowed to form a monolayer for 24 h. Following incubation, the cells were treated with varying concentrations of probiotic isolates (10^6^ to10^8^ CFU/mL) and cell-free extracts (filter-sterilized with a 0.2 μm filter). Untreated cells (medium only) were used as negative control, while cells treated with DMSO (Merck) served as positive control. After 24 h of treatment, the supernatant of each well was discarded. Next, 100 μL of 10% MTT (3-(4,5-dimethylthiazol-2-yl)-2,5-diphenyltetrazolium bromide) solution prepared in MEM was added to the wells, which were subsequently kept for 2–4 h at 37 °C in 5% CO_2._ After incubation, the MTT solution was discarded, a solubilization solution was added to dissolve the formazan crystals, and the absorbance was recorded at 492 nm.

#### Adhesion assay of the Caco-2 cell line

The adhesion ability of the isolates was assessed using the Caco-2 (human-derived colon carcinoma cell line) cell line following a previously described method (Rohith and Halami, 2021). In brief, 1 mL of 0.5 × 10^5^ cells/well were seeded in filter-sterilized MEM with 10% FBS in a 24-well plate followed by incubation at 37 °C for 24 h at 5% CO_2_. After incubation, the cells were monitored under an inverted microscope until they reached more than 80% confluence. 0.5 mL fresh MEM was added followed by 2 h incubation at 37 °C with 5% CO_2_. The isolates were cultured overnight (37 °C, 150 rpm) in brain heart infusion (BHI) broth followed by centrifugation at 1,000 rpm for 10 min. The obtained cell pellets were washed with sterile PBS containing 0.01% Tween 80 (pH 7.2) and resuspended in sterile PBS. Viable cell counts were determined using the pour plate method on TSA followed by incubation at 37 °C for 18–24 h. Colony counts were recorded and expressed as T0 (CFU/mL).

For the adhesion experiment, the cell pellet suspension was added to 0.5 mL of MEM to obtain a final concentration of 1 × 10^8^ CFU/mL/well. Incubation was done at 37 °C for 90 min. After the exposure period, the treated cell monolayers were separated via trypsinization (0.25% trypsin EDTA for 15 min at RT). The detached cells were scraped, and the liquid was aspirated into a pipette to loosen the cells from the plate surface (Shinde et al., 2019). The suspensions containing adherent bacteria attached to cell line were serially diluted in PBS supplemented with 0.01% Tween 80, plated onto TSA, and incubated at 37 °C for 24 h to enumerate viable count. The recovered bacteria were expressed as T1 (CFU/mL). The percentage (%) adhesion score was calculated as (T1/T0) *100. Here, T0 represents the initial viable count of isolates prior to exposure to the Caco-2 monolayer, whereas T1 represents the number of cells that remained adherent to the Caco-2 cells after incubation.

#### Inhibition of pathogenic bacterial adhesion to Caco-2 cells (invasion assay)

Pathogenic *E. coli* ATCC8739 inhibition by isolates from the colonizing Caco-2 cell line was measured via an invasion assay with modifications (Rohith and Halami, 2021). For the competition assay*, Bacillus* isolates (1 × 10^8^ CFU/mL) and *E. coli* (1 × 10^8^ CFU/mL) were added to MEM (without antibiotics and FBS), suspended in Caco-2 cells followed by incubation at 37 °C for 90 min in 5% CO_2_. Post-incubation, non-adhered bacterial cells were detached by washing the wells three times with PBS. Cells that remained attached were recovered by treating the monolayer with 0.25% trypsin at 37 °C for 10 min. The detached bacterial cells were plated in their respective media (TSA for *Bacillus* and brain heart infusion agar for *E. coli*), and the obtained bacterial count was expressed in CFU/mL. Control or untreated wells were maintained for both *Bacillus* isolates *and E. coli* as standard for all the assays.

For the exclusion assay, *Bacillus* (1 × 10^8^ CFU/mL) was introduced into the Caco-2 cell line followed by incubation at 37 °C for 90 min in 5% CO_2_. Weakly attached bacterial cells were detached by thorough washing in PBS followed by addition of *E. coli* (1 × 10^8^ CFU/mL) and incubation at 37 °C for 90 min in 5% CO_2_. After completion of incubation, the weakly adhered cells were detached by washing in PBS, and the adhered bacterial cells were obtained via trypsinization followed by plating in the appropriate medium.

For the displacement assay, *E. coli* (1 × 10^8^ CFU/mL) was initially introduced into Caco-2 cells followed by subsequent incubation at 37 °C for 90 min in 5% CO_2_. Weakly adhered bacterial cells were detached by washing in PBS. After washing, *Bacillus* (1 × 10^8^ CFU/mL) was added to Caco-2 cells. Subsequent incubation, washing, recovery, and enumeration were performed as described for the exclusion assay.

#### Enzyme production

The production of extracellular enzymes by the isolates was evaluated using the substrate hydrolysis assay. The specific media plates were supplemented with the respective substrates. Starch (HiMedia) for amylase, casein 0.5% w/v for protease, tributyrin (HiMedia) for lipase, and sodium phytate (HiMedia) for phytase were used (Mussa et al., 2023; Sreenadh et al., 2022; Vijayaraghavan and Vincent, 2013). For beta-galactosidase, ONPG (O-nitrophenyl-*β*-D-galactopyranoside) broth (HiMedia) was used (Lata et al., 2022). In brief, a test inoculum (~1.00 OD) was spot inoculated (20 μL) on the respective media plate followed by incubation at 37 °C for 24–72 h. For amylase, the plate was flooded with 0.1% iodine solution to visualize the zone. For protease, lipase and phytase clear zones around the colonies represent the hydrolysis of the substrate through the enzyme. The enzyme activity was assessed by measuring the clear hydrolysis zone diameter (in mm) to the colony diameter, and the results were presented as zone of inhibition.

For beta-galactosidase, 0.1 mL of cultured broth was added to 4.9 mL of filter-sterilized ONPG broth followed by incubation at 37 °C for 24 h. β-galactosidase activity was confirmed by development of yellow coloured broth due to the release of o-nitrophenyl. An uninoculated tube used as a control and a tube incubated with *Salmonella enterica* ATCC® 14,028 were treated as a negative control.

#### Resistance to simulated gastric and intestinal conditions

Tolerance of the isolates to simulated gastric and intestinal conditions was examined using *in vitro* model adapted from the previously described protocol (Mazhar et al., 2024; Toutiaee et al., 2022). In brief, 1 mL of spore suspension (>1×10^9^ CFU/mL) was added to 9 mL of PBS (pH 3) supplemented with 0.3% pepsin (HiMedia, India). The suspension was incubated at 37 °C under shaking for 2 h to mimic gastric conditions. Following exposure, cells were recovered by centrifugation (10,000 rpm for 10 min), washed in PBS, and resuspended in 10 mL of PBS (pH 7.5) containing 0.1% (w/v) pancreatin (HiMedia, India) and 0.3% (w/v) bile salt (HiMedia, India). The mixture was incubated for 4 h at 37 °C with shaking to simulate intestinal condition. After incubation, samples were collected, serially diluted, and plated on TSA agar at intervals of 1 and 2 h for gastric phase and 2 and 4 h for intestinal phase. The percentage survival was calculated via T*_treatment_*/T*_initial_* * 100, Where T initial and T treatment represent the numbers of surviving bacteria (CFU/mL) before and after treatment, respectively.

#### Antioxidant activity

##### DPPH assay

The antioxidant potential of the isolates was evaluated via previously described methods with minor changes (Lee et al., 2017; Khan et al., 2023). In brief, 2 mL of 0.4 mM 2,2-diphenyl-1-picrylhydrazyl (DPPH) (Merck) prepared in methanol was added in 2 mL of overnight-grown bacterial culture (OD of 0.5–1 with ~1 × 10^8^ CFU/mL) in a sterile tube. The reaction mixture was incubated in dark for 30 min at 37 °C. As a positive control, 1 mg/mL ascorbic acid was used for comparison. After 30 min of incubation, the mixture was centrifuged at 8,000 rpm for 10 min at 4 °C and the absorbance of the supernatant was measured at 517 nm. The antioxidant activity was calculated using the formula 1- [Absorbance of test/Absorbance of blank] *100.

##### Hydroxyl radical scavenging activity

This attribute is a measure of the ability of substances that can neutralize hydroxyl ions (–OH ions). The antioxidant activity of the isolates was evaluated via the modified phenanthroline-based assay as previously described (Chen et al., 2014; Mukhopadhyay et al., 2016). Then, 1 mL of 2.5 mM FeSO_4_ (SD Fine), 1 mL of PBS (pH 7.5), 1 mL of intact cells, and CFS were mixed. The reaction was initiated by adding 1 mL of 20 mM H_2_O_2_ (Qualigen) and 1 mL of 2.5 mM 1,10-phenanthroline (Merck). The mixture was incubated at 37 °C for 90 min. The absorbance was recorded at 536 nm using UV–Vis spectrophotometer (Shimadzu UV-1900i). Distilled water was taken as the reagent blank and sample control. Scavenging activity was measured as follows:

Scavenging effect (%) = [A_536_ (Sample) − A_536_ (Blank)]/[A_536_ (Control) − A_536_ (Blank)] × 100 (Giri et al., 2019).

#### Biogenic amine-producing capacity

Biogenic amines (BAs) are toxic compounds that can be produced by certain bacteria and can cause nausea, respiratory distress, hot flashes, etc. (Chang and Chang, 2012). For the screening of biogenic amine (BA) producers and non-producers, a modified broth method was employed (Lee et al., 2017; Chang and Chang, 2012). In brief, isolates were grown overnight in LB at 37 °C with shaking, and 1 mL of each overnight culture was inoculated into 9 mL of LB-based screening broth media supplemented with 0.006% bromocresol purple (Merck) at pH 5.0. The screening media included LB broth (as a control), LB with glycerol (0.25%) (SD FCL) (LB-GL), LB with glycerol (0.25%) and ornithine (0.1%) (Merck) (LB-GLO), and LB with glycerol (0.25%) and histidine (0.1%) (SD FCL) (LB-GLH). Incubation was done at 37 °C for 24 h with shaking. Following incubation, the cells were centrifuged at 10,000 rpm for 15 min at 4 °C, and the supernatants were collected. The pH values of the supernatant were measured, and colour development was quantified at 590 nm via a UV–Vis spectrophotometer (Shimadzu UV-1900i). LB broth with bromocresol purple without culture was treated as the control. In BA negative but glycerol metabolizing isolates, acidic by-products formed during glycerol metabolism counterbalance alkaline compounds, resulting in the retention of a yellow colour in the medium. In contrast, isolates capable of both glycerol metabolism and BA production generate excess alkaline metabolite when amino acids are present. In such cases, the acids produced are insufficient to neutralize the additional alkalinity, leading to a purple coloration of the medium (Chang and Chang, 2012).

### Statistical analysis

All data were expressed as means ± standard errors of the means (SEMs). Statistical analyses were performed via GraphPad Prism version 10.5.0. Comparisons were performed using two-way analysis of variance (ANOVA) followed by suitable *post-hoc* tests. Differences were considered statistically significant when the *p*-value was <0.05.

## Results and discussion

### Isolation and identification

A total of 120 ethically sourced meconium samples collected from healthy newborns were screened, resulting in the isolation of 171 bacterial strains. All the isolates were initially characterized by Gram staining, of which 167 were identified as spore-forming bacteria and subsequently evaluated for their probiotic potential. From this pool, three strains demonstrating superior functional attribute were selected and designated as ZBOE6, ZBRV8, and ZBTM2.

Identification—Whole-genome sequencing followed by annotation and genome-based taxonomic analysis confirmed all three isolates as *Bacillus subtilis.* ZBOE6 had a draft genome of 4,306,554 bp (GC content 43.35%), ZBRV8 of 4,051,041 bp (GC content 43.52%), and ZBTM2 of 4,048,501 bp (GC content 43.5%). CheckM analysis indicated high genome completeness (99.81%) with low contamination (<0.4%) across all assemblies, supporting reliability of downstream genomic interpretation. Taxonomic assignment showed gyrB sequence identity >99.8% to *Bacillus subtilis* strains, ANI > 98% to *Bacillus subtilis sub*sp. *Subtilis,* and TYGS dDDH values of 92.9% (ZBOE6), 87.9% (ZBRV8), and 87.9% (ZBTM2) relative to *Bacillus subtilis* ATCC 6051. These values exceed established species delineation thresholds, confirming all three isolates are the member of *Bacillus subtilis*. The corresponding genome assemblies were deposited in the NCBI GenBank database, and their accession numbers are presented in Table 1.

**Table 1:** Identification of isolates by whole-genome sequencing along with accession number.

Isolate no.	Taxonomic identification	Whole-genome accession number
ZBOE6	*Bacillus subtilis*	JBDMAP000000000
ZBRV8	*Bacillus subtilis*	JBNFZN000000000
ZBTM2	*Bacillus subtilis*	JBNFMW000000000

The sequence was deposited in NCBI GenBank and is openly accessible.

#### Genomic basis of probiotic traits

The genomes of all three isolates exhibited high completeness and encoded a broad repertoire of gene associated with probiotic functionality (Table 2). However, beyond shared core features, comparative analysis revealed subtle but functionally relevant strain-specific differences that may explain the observed phenotypic variability. Genes associated with acid tolerance including *perR*, *fur*, *nsrR*, and *sigB*, (11–13 genes) along with bile salt hydrolase (*Choloylglycine hydrolase* EC 3.5.1.24) were present across all strains, supporting their ability to survive gastrointestinal stress conditions. Notably, stress regulator *sigB* was present in ZBOE6 and ZBTM2 but absent in ZBRV8 which may contribute to differences in stress resilience observed during *in vitro* assays. Core regulators such as *perR* and *fur*, known to co-ordinate oxidative and acid stress responses, further support survival under simulated gastrointestinal conditions.

**Table 2:** Genome-based screening of probiotic-associated features across three isolates.

Functional categories	ZBOE6 (key genes)	ZBRV8 (key genes)	ZBTM2 (key genes)	Biological relevance
Acid tolerance	12 (*perR, fur, nsrR, sigB*)	11 (*perR, fur, nsrR*)	13 (*perR, fur, nsrR, sigB*)	Gastric transit survival
Bile tolerance/BSH	1 (*Choloylglycine hydrolase EC 3.5.1.24*)	1 (*Choloylglycine hydrolase*)	1 (*Choloylglycine hydrolase*)	Bile salt deconjugation
Oxidative stress	17 (sodA, sodC, sodMn, gpx, kat, tpx, bcp)	15 (sodA, sodC, gpx, kat, tpx)	16 (*sodA, sodC, sodMn, gpx, bcp*)	ROS neutralization in gut
Osmotic stress	9 (*opuAA, opuAB, opuAC, opuD, gbsB*)	8 (*opuAA, opuAB, opuD, gbsB*)	9 (*opuAA, opuAB, opuAC, gbsB*)	Intestinal hypertonicity
Sporulation	28 (*spo0A, spoIIE, spoIIR, sigF, sigE, sigG, sigH, sigK*)	26 (*spo0A, spoIIE, sigF, sigE, sigG*)	27 (*spo0A, spoIIE, sigF, sigE, sigG, sigK*)	Spore formation resilience
Spore germination	9 (*gerPA, gerPB, gerPC, gerPD, sleB, cwlJ*)	8 (*gerPA, gerPB, gerPC, sleB*)	9 (*gerPA, gerPB, gerPC, gerPD, sleB*)	Spore germination
Biofilm/EPS	6 (*tasA, sipW, yqxM, sinI, sinR, slr*)	5 (*tasA, sipW, yqxM, sinR*)	6 (*tasA, sipW, yqxM, sinI, sinR*)	Adhesion/colonization
Carbohydrate utilization	24 (*bglA, bglH, amyE, sacB, licT, iol cluster*)	22 (*bglA, amyE, sacB, iol cluster*)	23 (*bglA, bglH, amyE, licT*)	Prebiotic metabolism
Probiotic metabolism	12 (*butB, acuA/B/C, acoA/B, riboflavin cluster*)	11 (*butB, acuA/B, acoA/B*)	12 (*butB, acuA/B/C, riboflavin cluster*)	Functional compound production

Oxidative stress defence included multiple superoxide dismutase’s (*sodA/sodC/sodMn*), catalases (*kat*), and peroxiredoxins (*tpx*, *bcp*; 15–17 genes). ZBOE6 exhibited the highest number of oxidative stress genes (Ozkan et al., 2013) followed by ZBTM2 (Ben Taheur et al., 2016) and ZBRV8 (Myintzaw et al., 2023), suggesting a relatively enhanced capacity for reactive oxygen species detoxification in ZBOE6, which may explain partially its relatively higher antioxidant activity observed *in vitro*. The presence of key enzyme such as catalase (*KatA*) and superoxide dismutase (*sodA*) across all strains supports a conserved antioxidant defence system. Osmotic stress adaptation was supported by genes involved in glycine betaine uptake including *opuAA, opuA, opuAC, opuD,* and *gbsB* (8–9 genes). ZBTM2 displayed slightly enriched osmotic stress gene repertoire, which may be associated with its comparatively higher bile tolerance observed experimentally, given the role of osmo-protectant systems in maintaining cellular homeostasis under bile induced stress. Sporulation pathways were largely complete in all strains, including key regulators such as *spo0A*, *sigF, sigE, sigG, sigH, sigK*, and *spoIIE/R* (26–28 genes), along with germination-associated clusters (*gerPA/B/C/D*, *sleB*; 8–9 genes). These pathways ensure environmental resilience and efficient transition between dormant and vegetative states, supporting persistence during gastrointestinal transit across all the strains.

Genes associated with biofilm formation and colonization including *tasA*, *sipW*, *yqxM*, and *sinR/I* (5–6 genes) were identified in all the strains. ZBOE6 and ZBTM2 possess a more complete regulatory set, including *sinl* which was absent in ZBRV8, potentially contributing to differences in adhesion and aggregation observed *in vitro*. The presence of *tasA, sipW, and yqxM* operon supports extracellular matrix production and cell-surface interaction supporting a mechanistic basis of adhesion-related phenotypes. Carbohydrate utilization pathways, including *bglA*, *bglH, amyE, sacB, licT,* and *iol* cluster (22–24 genes), were broadly distributed across the strains. ZBOE6 exhibited the most diverse carbohydrate metabolism gene set, which may provide a competitive advantage under nutrient variable conditions. Genes associated with probiotic metabolite production, including *butB*, *acuA/B/C*, *acoA/B*, and riboflavin biosynthetic cluster (11–12 genes), were also identified. ZBTM2 retained a complete riboflavin biosynthesis cluster, while ZBRV8 showed a relatively reduced metabolic gene set, which may influence its functional performance under stress conditions. In addition, biosynthetic gene cluster encoding antimicrobial compound such as surfactin, fengycin, bacillibactin, bacilysin, and subtilosin A were identified, with ZBRV8 showing relatively prominent representation. These metabolites are known to exert antimicrobial activity and competitive exclusion, although the limited antimicrobial activity observed *in vitro* suggests that the expression of these clusters may be condition-dependent. Overall while the genomic data provide mechanistic insight into potential probiotic traits, it is important to note that the presence of gene does not necessarily indicate functional expression under *in vivo* conditions. Therefore, future transcriptomic, proteomic, and *in vivo* studies are required to validate the strain-specific functional attributes.

#### Safety assessment of the isolates

Assessment of safety-related traits is a critical pre-requisite for probiotic, requiring both phenotypic and genome-based screening to exclude pathogenic potential and transferable resistance. Non-haemolytic and DNase negative traits are widely accepted indicators of probiotic strain as non-pathogenic and non-toxic. As shown in Table 3, *in vitro* analysis revealed strain-specific haemolytic patterns, with ZBOE6 exhibiting α haemolysis (partial hydrolysis/green zone), while ZBRV8 and ZBTM2 showed γ (no hydrolysis) haemolysis. As per previous reports, α and γ haemolysis are considered safe, whereas β haemolysis (clear zone) is considered harmful (Lee et al., 2017). Genomic-based screening further supported these observations as no pathogenic haemolysin genes (hlyA/B/C, cereolysin O) were detected in ZBOE6, ZBRV8, and ZBTM2 via ABRicate (NCBI/VFDB; gyrB control: 99.8–100% identity for housekeeping controls). Only hypothetical ‘yplQ Haemolysin-3-like protein’ annotated in all three strains via Bakta v1.9.3, which is responsible for membrane fluidity and unsaturated fatty acid regulation (Melchionna et al., 2022). α phenotype of ZBOE6 correlates with safe H_2_O_2_-mediated lysis (lactate oxidase present). Combined phenotypic and genomic data confirmed the strains non-haemolytic potential, supporting their safety for probiotic applications.

**Table 3:** Result of haemolytic activity and DNase activity of isolates.

Isolate no.	Haemolytic activity	DNase activity
ZBOE6	*α*	Negative
ZBRV8	*γ*	Negative
ZBTM2	*γ*	Negative

DNase activity was absent in all strains, as indicated by the absence of a clear zone around the colonies (Sreenadh et al., 2022). This phenotype is consistent with genomic data, which did not identify genes associated with extracellular DNase-mediated virulence, further supporting the non-pathogenic nature of the isolates. Collectively, the phenotypic assays and genomic analysis strengthen the safety profile of the strains, indicating of key virulence determinants. However, these findings are *in vivo* and *in silico* assessments and should be further validated under *in vivo* conditions.

#### Biofilm formation

The formation of biofilms is one of the desired attribute for prospective probiotics as it suggests mucosal attachment and persistence within the gastrointestinal tract (Hong et al., 2008). All three isolates demonstrated the ability to retain crystal violet, suggesting biofilm-forming capacity under *in vitro* conditions. These findings align with earlier reports where isolates retained crystal violet, confirming to establish stable colonization and provide a competitive barrier against pathogens in the host (Ozkan et al., 2013). While biofilm formation enhances colonization efficiency, it can also possess critical risk for horizontal gene transfer of antibiotic resistance gene or be associated with increased virulence. *In silico* analysis confirmed the absence of acquired antibiotic resistance genes and known virulence factors. These findings suggest that the biofilm-forming capacity of the isolates is a functional trait for stable host colonization and does not coincide with pathogenic potential.

#### Acid and bile tolerance

To confer health benefits on the host, a probiotic should be tolerant to the strong acidic conditions of the stomach and the bile-rich environment of the intestinal (Lee et al., 2017). Orally ingested bacteria must remain viable during transit through unfavourable conditions before reaching the large intestine (Rohith and Halami, 2021). In this study, all three strains exhibited substantial acid tolerance, with ZBOE6 showing the highest survival (91.09 ± 5.42%), followed by ZBRV8 (85.76 ± 8.65%) and ZBTM2 (78.65 ± 3.23%).

**Figure 1:**
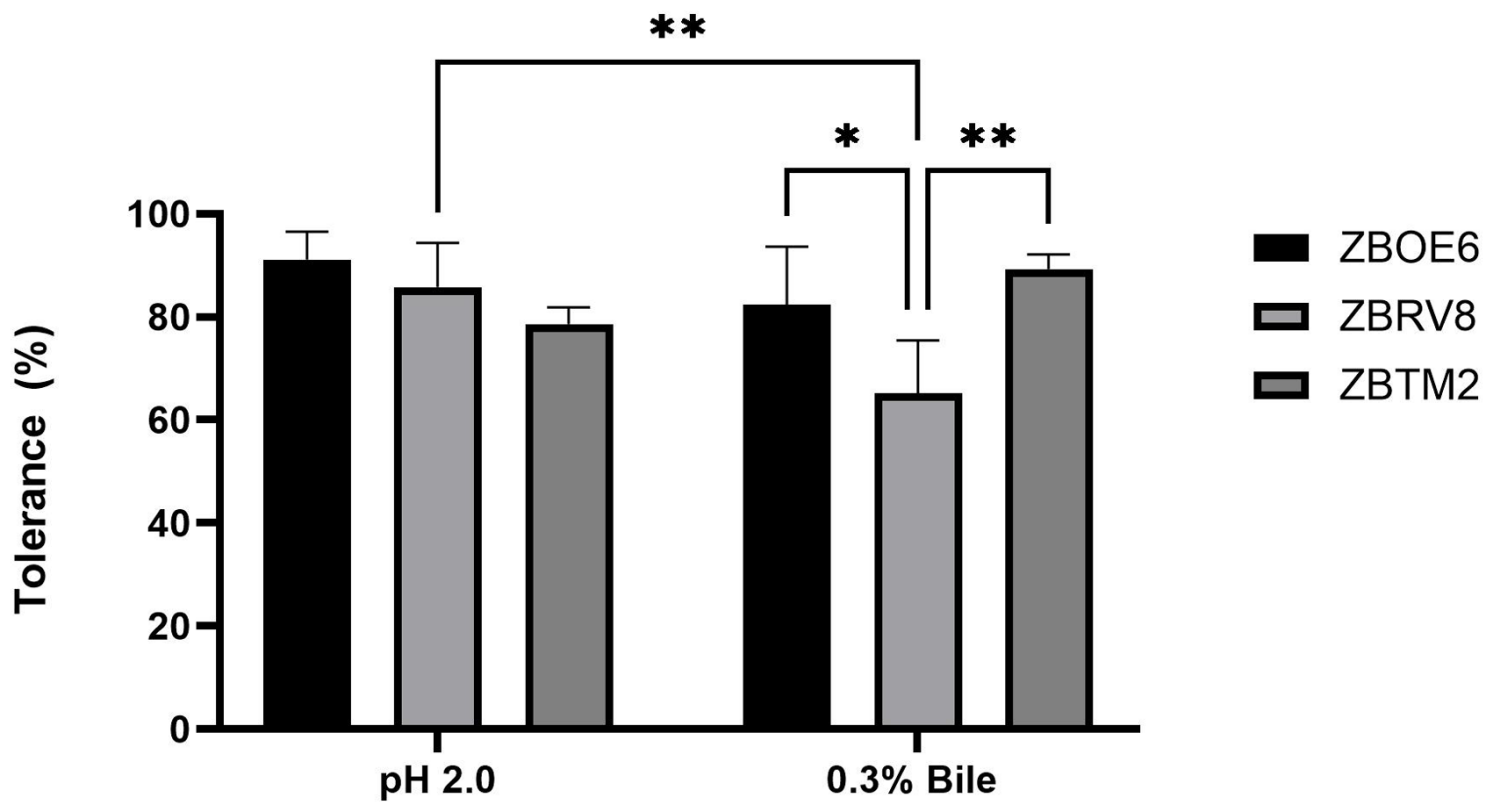
Acid and bile tolerance (%) of isolates ZBOE6, ZBRV8, and ZBTM2 after exposure to pH 2 for 90 min and 0.3% bile. Data are presented as the mean ± SD (*n* = 3). No significant differences were observed under acidic conditions, whereas under bile stress ZBRV8 showed significantly lower tolerance than ZBOE6 (^*^p < 0.05) and ZBTM2 (^**^p < 0.01), with a significant reduction in survival between acid and bile conditions observed only for ZBRV8 (^**^p < 0.01).

For bile tolerance, quantified using a CFU-based survival assay, ZBTM2 demonstrated the highest survival (89.32 ± 2.77%), followed by ZBOE6 (82.36 ± 11.28%) and ZBRV8 (65.16 ± 10.27%), indicating robust but strain-dependent tolerance under bile stress conditions. These strain-specific differences are consistent with genomic stress response features identified across all the strains. In particular, the presence of stress regulators (*sigB, perR, and fur*) and acid tolerance-associated enzymes (glutamate-5-kinase) may contribute to intracellular pH homeostasis and survival under acidic conditions. Similarly, the relatively higher bile tolerance observed in ZBTM2 may be associated with its osmotic stress-related gene repertoire, including components of the *opu* operon, which are known to facilitate cellular homeostasis under stress condition. Overall, these findings suggest that the isolate may possess the capacity to withstand gastrointestinal stress conditions, although *in vivo* validation is required to confirm their survival and functional persistence in the host environment (Figure 1).

#### Bile salt hydrolase (BSH) activity

BSH activity is an important functional attribute of probiotic strains due to its role in bile salt metabolism and its potential influence on host lipid homeostasis (Majeed et al., 2019). The precise mechanism remains to be elucidated, and BSH enzyme produced by gut bacteria deconjugates bile acid into their free forms, which may be subsequently eliminated via faecal excretion (Kumar et al., 2012). All three strains demonstrated the ability to hydrolyse TDC, as evidenced by deoxycholate precipitation on the assay medium. Genomic annotation (Bakta v1.9.3, RAST) confirmed the presence of *Bsh* gene encoding choloylglycine hydrolase (EC 3.5.1.24; Ntn-hydrolase superfamily) across all the strains within bile hydrolysis subsystems, consistent with the observed phenotypic activity. Both this phenotypic and genomic evidence supports the functional potential of these strains in bile salt metabolism. However, the contribution of BSH activity to cholesterol reduction remains indirect and context-dependent; therefore, these findings should be interpreted as indicative property of cholesterol assimilation potential rather than a confirmed cholesterol lowering effects. Further *in vivo* studies are required to validate this functional role of the strains.

#### Auto-aggregation and co-aggregation

**Figure 2:**
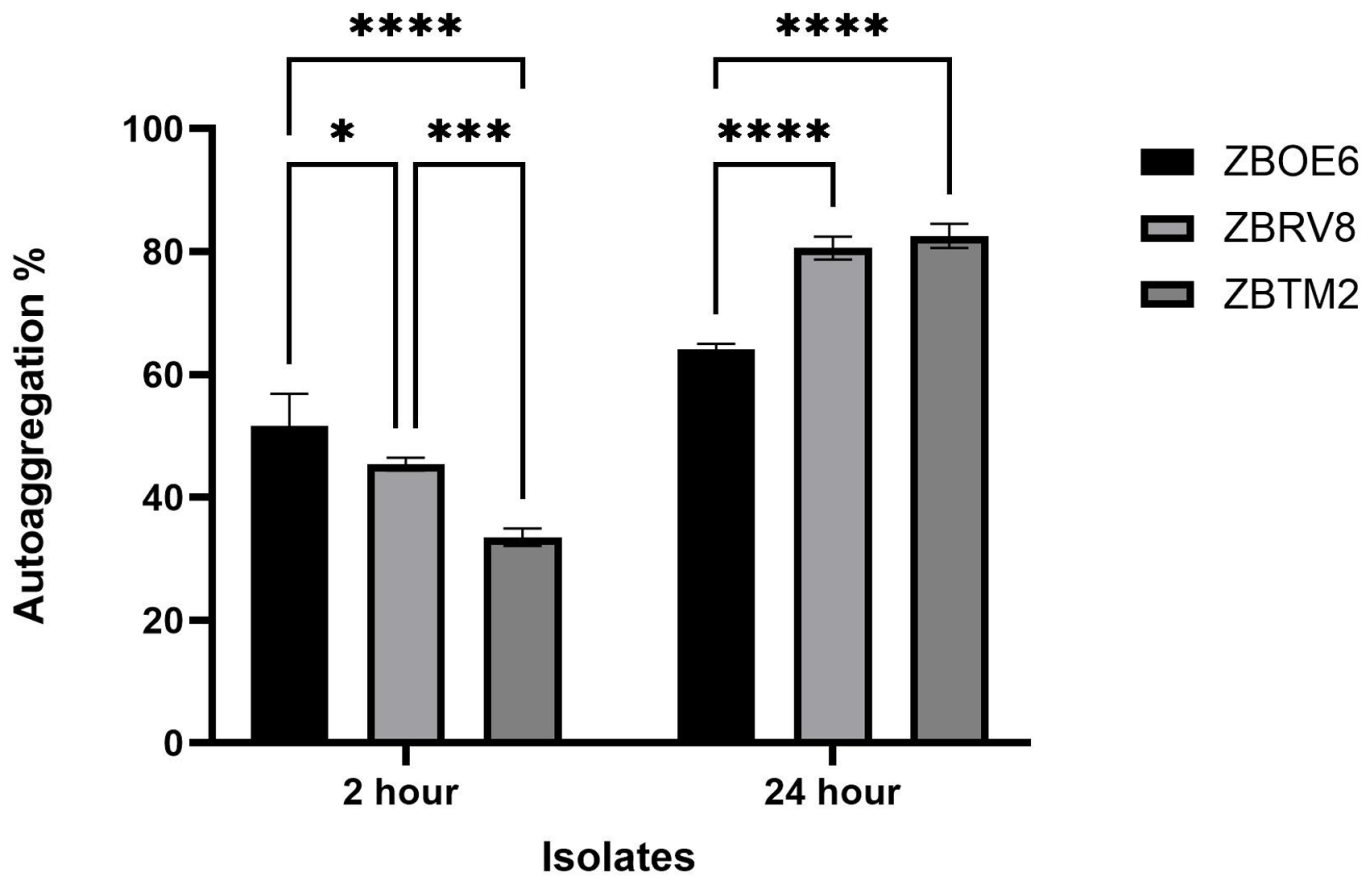
Percentage auto-aggregation of isolates ZBOE6, ZBRV8, and ZBTM2 at 2 h and 24 h. Data are presented as mean ± SD (*n* = 3). *p*-value ≤ 0.05. ^*^ < 0.05; ^***^ < 0.001; ^****^p < 0.0001.

Auto-aggregation is an important property that reflects the ability of probiotic strains to self-associate and form stable microbial communities, which can facilitate colonization of gastrointestinal tract. Strains auto-aggregation assays were conducted at 2 h and 24 h, and the results are shown in Figure 2.

ZBOE6 exhibited strong initial auto-aggregation (51.65 ± 5.25%) at 2 h, followed by ZBRV8 (45.39 ± 1.06%) and ZBTM2 (33.52 ± 1.44%). After 24 h under static conditions, a significantly greater aggregation percentage was observed with ZBTM2 (82.58 ± 1.98%), closely followed by ZBRV8 (80.59 ± 1.85%). ZBOE6 exhibited a moderate increase of 64.05 ± 0.93%. Overall, all the strains exhibited a good auto-aggregation after 24 h, suggesting intestinal colonization potential. According to previous studies, more than 40% is considered as good auto-aggregation ability whereas strains having less than 10% activity is considered as weak auto-aggregation (Mazhar et al., 2024).

**Figure 3:**
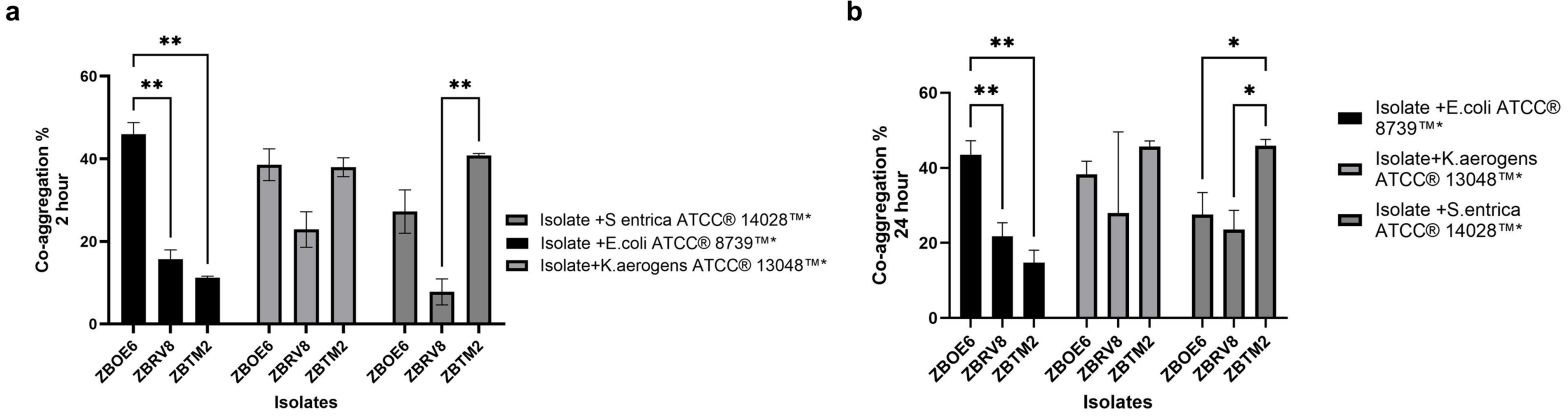
Co-aggregation abilities of isolates with pathogenic strains at 2 h **(a)** and 24 h **(b)**. Bars represent the mean percentage of co-aggregation (± SD) between isolates (ZBOE6, ZBRV8, and ZBTM2) and *E. coli* ATCC® 8,739™, *S. enterica* ATCC® 14,028™, and *K. pneumoniae* ATCC® 700,603™. Statistical significance is denoted as *p* < 0.05 (*), *p* < 0.01.

The co-aggregation ability (Figure 3) of the isolates was evaluated with pathogenic strains after 2 h (Figure 3a) and 24 h (Figure 3b) (Jeon et al., 2017; Collado et al., 2008). ZBOE6 exhibited greater co-aggregation ability with *E. coli* ATCC® 8,739™ at both time points (45.97 ± 2.23% at 2 h; 43.54 ± 3.28% at 24 h), which was significantly greater than that of ZBRV8 and ZBTM2. ZBTM2 strongly co-aggregated with *Salmonella enterica* ATCC® 14,028™ (40.86 ± 0.25% at 2 h; 45.88 ± 2.52% at 24 h).

*K. aerogenes* ATCC® 13,048™ did not significantly differ among strains at any time point. Overall, the selected isolates exhibited good co-aggregation ability with pathogenic bacteria, indicating potential competitive exclusion mechanism against pathogens. The mechanisms underlying aggregation are multifactorial involving surface hydrophobicity, pili, adhesion, etc. which can be further studied using genetic and proteomic approaches. Together, auto-aggregation and co-aggregation provide an advantage to the probiotic strain for microbial colonization in the intestinal tract.

#### Microbial adhesion to hydrocarbons

**Figure 4:**
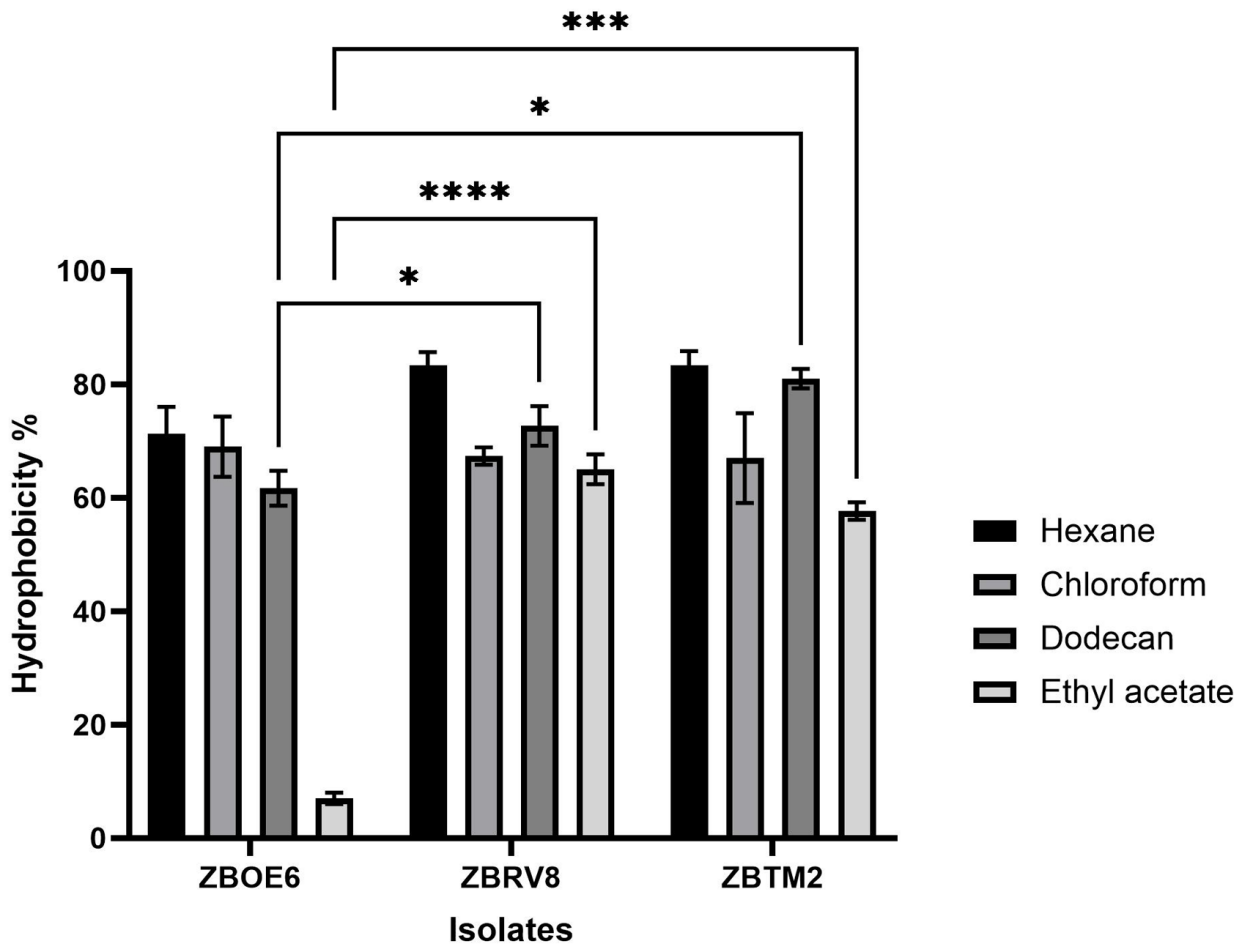
MAHT (%) of the isolates ZBOE6, ZBRV8, and ZBTM2 assessed using different solvents: hexane, chloroform, dodecane, and ethyl acetate. Data are denoted as the mean ± SD (*n* = 3). Significant variation among the isolates was detected in their interaction with dodecane (*p < 0.05) and ethyl acetate (^**^p < 0.001, ^***^p < 0.0001).

This assay is used for the assessment of adherence ability of probiotics to adhere to intestinal cells (Krausova et al., 2019). Higher hydrophobicity is considered advantageous for probiotics as it enhances their capacity to adhere to human intestinal epithelial cells (Shivangi et al., 2020). This property facilitates the initial interaction between the microorganisms and the intestinal epithelium of the host (De Souza et al., 2019). Previous studies have shown that adhesion to hexadecane (non-polar) reflects the overall hydrophobic/hydrophilic nature of the bacterial surface, and affinity towards chloroform (polar acidic solvent) and ethyl acetate (polar basic solvent) indicates the electron donor and electron acceptor properties of the bacterial cell surface, respectively (Lim et al., 2021). All the isolates showed strong hydrophobic interactions (Figure 4), with values of 71.3 ± 4.7% (ZBOE6), 83.3 ± 2.3% (ZBRV8), and 83.3 ± 2.5% (ZBTM2) with hexane, indicating that the hydrophobic surfaces of the isolates were similar. Chloroform also exhibited a similar pattern (*p* > 0.05), with values of 69.0 ± 5.3% (ZBOE6), 67.3 ± 1.5% (ZBRV8), and 67.0 ± 6.1% (ZBTM2). In contrast, ZBTM2 (81.0 ± 1.7%) and ZBRV8 (72.7 ± 3.2%) were more hydrophobic than ZBOE6 (61.7 ± 3.1%) (*p* < 0.05) with dodecane, while with ethyl acetate, ZBRV8 (65.0 ± 2.6%) and ZBTM2 (57.7 ± 1.5%) presented significantly higher MAHT values than ZBOE6 (7.0 ± 1.0%). Overall, all the selected isolates were relatively hydrophobic, indicating a comprehensive profile of surface characteristics that may influence adhesion.

#### Antibiotic susceptibility

The strains were also evaluated for sensitivity to clinically relevant antibiotics using cutoff values suggested for probiotic safety evaluations. Antibiotic susceptibility of probiotic strains is crucial for safety as resistance genes may be transferred via plasmids, transposons, or chromosomal mutations, potentially contributing to the emergence of new resistant strains (Jeon et al., 2017). Isolates were assessed for sensitivity to eight clinically relevant antibiotics via cutoff values suggested for probiotic safety evaluation for *Bacillus* sp. As shown in Table 4, ZBOE6 was resistant to tetracycline (32.00 ± 0.00 μg/mL), whereas ZBRV8 was resistant to streptomycin (21.33 ± 4.62 μg/mL) and clindamycin (1.83 ± 0.29 μg/mL), with MIC values exceeding the cutoff thresholds. ZBTM2 was sensitive against all the antibiotics, with MICs well within the EFSA thresholds. Similar findings have been reported for *Bacillus subtilis* strains such as KATMIRA1933 and *B. amyloliquefaciens* B-1895 (AlGburi et al., 2016). Genomic investigation of isolates ZBOE6, ZBRV8, and ZBTM2 confirmed the absence of transferable or high-risk antibiotic resistance genes in their whole-genome sequences. ZBOE6 and ZBRV8 annotation and antimicrobial resistance screening identified only a limited set of resistance-associated chromosomal loci, all intrinsic and not linked to mobile genetic elements such as plasmids, transposons, or genomic islands. No genes associated with horizontal gene transfer or clinically significant resistance were detected, and all the isolates lacked active virulence genes and insertion sequence-associated mobile elements. This finding aligns with the European Food Safety Authority Presumption of safety (QPS) criteria for *Bacillus subtilis*, which requires the absence of acquired resistance in clinically relevant antimicrobials (EFSA Panel on Biological Hazards (BIOHAZ) et al., 2026). The antibiotic resistance in phenotypic testing is likely due to intrinsic mechanisms and not due to the presence of transferable resistance determinants, supporting the genomic safety profile of these isolates. The genomic safety profile can further be validated through animal or human trials when considered for clinical development.

**Table 4:** Antibiotic susceptibility profile of isolates based on MIC values (μg/mL).

Isolate(cutoff value in ppm)	ZBOE6	ZBRV8	ZBTM2
Vancomycin (4)	1.67 ± 0.29 (S)	2.67 ± 0.58 (S)	1.33 ± 0.29 (S)
Gentamicin (4)	0.38 ± 0.00 (S)	0.42 ± 0.07 (S)	0.83 ± 0.14 (S)
Kanamycin (8)	1.50 ± 0.00 (S)	0.46 ± 0.07 (S)	0.75 ± 0.25 (S)
Streptomycin (8)	3.33 ± 0.58 (S)	21.33 ± 4.62 (R)	6.00 ± 0.00 (S)
Erythromycin (4)	0.75 ± 0.25 (S)	0.23 ± 0.03 (S)	0.83 ± 0.14 (S)
Clindamycin (4)	0.38 ± 0.00 (S)	1.83 ± 0.29 (R)	0.67 ± 0.14 (S)
Tetracycline (8)	32.00 ± 0.00 (R)	0.92 ± 0.14 (S)	0.46 ± 0.07 (S)
Chloramphenicol (8)	4.67 ± 2.31 (S)	2.33 ± 0.58 (S)	5.33 ± 1.15 (S)

The susceptibility interpretation (S, sensitive, R, resistant) was determined using EFSA recommended cutoff values. Data are presented as the mean ± standard deviation (*n* = 3).

#### Antimicrobial activity against pathogens

The antimicrobial properties of all the isolates were measured against six pathogenic strains using an agar well diffusion assay (Table 5).

**Table 5:** Antimicrobial activity of isolates (ZBOE6, ZBRV8, and ZBTM2) against selected pathogens assessed by agar well diffusion assay.

Pathogen	ZBOE6		ZBRV8		ZBTM2	
	CFE	Culture (OD1)	CFE	Culture (OD1)	CFE	Culture (OD1)
*E. coli* ATCC® 8,739™	nd	nd	Nd	nd	Nd	nd
*S. typhimurium* ATC® 14,028™	nd	nd	Nd	nd	Nd	nd
*K. aerogens* ATCC13048	nd	nd	Nd	nd	nd	nd
*C. albicans* ATCC® 10,231™	1.38 ± 0.03	nd	Nd	nd	nd	nd
*S. aureus* ATCC® 6,538	nd	nd	Nd	nd	nd	nd
*P. aeruginosa* ATCC® 9,027™	nd	nd	Nd	nd	nd	nd

Values represent zone of inhibition (mm) expressed as the mean ± SD (*n* = 3). “nd” indicates no detectable inhibition.

None of the isolates inhibited *Escherichia coli* ATCC® 8,739™, *Salmonella typhimurium* ATCC® 14,028™, *Klebsiella aerogenes* ATCC® 13,048, *Staphylococcus aureus* ATCC® 6,538, or *Pseudomonas aeruginosa* ATCC® 9,027™, as indicated by the absence of inhibition zones (nd). The cell-free extract (CFE) of strain ZBOE6 demonstrated weak antifungal activity against *C. albicans* ATCC® 10,231™, with a zone of inhibition measuring 1.38 ± 0.03 mm. This finding suggests that antimicrobial effects, if present, are not limited to production of diffusible compounds (e.g., bacteriocins) under the tested *in vitro* conditions. Similar findings have been reported where *Bacillus subtilis* has been shown to exhibit limited or no antibacterial activity against multiple pathogenic strains (Duc et al., 2004). Yet, another study reported no detectable antimicrobial activity against several gram-positive and gram-negative pathogens for the well-characterized *Bacillus subtilis* strain HU58, which is used commercially as probiotic for human use (Hong et al., 2009). These findings also suggest that these isolates may not exert antagonistic effects through secreted bacteriocins or lytic enzymes but may rely on cell interactions, such as co-aggregation, biofilm formation, adhesion to intestinal epithelium, and surface exclusion.

#### Cholesterol assimilation

**Figure 5:**
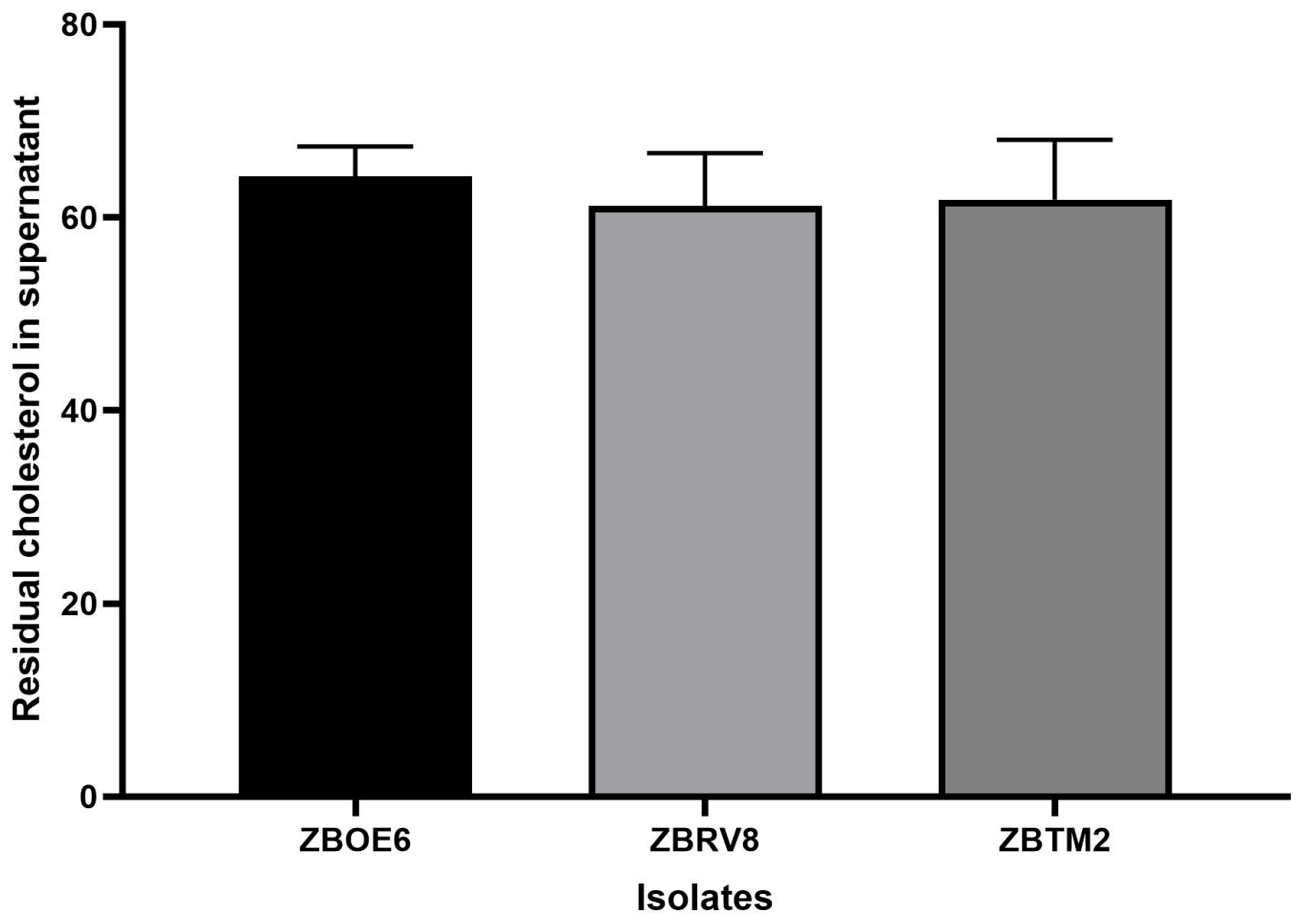
Residual cholesterol concentration in the cell-free supernatant of isolates ZBOE6, ZBRV8, and ZBTM2 after 18 h of incubation in LB broth containing 100 μg/L cholesterol. The results are expressed as the mean ± SD (*n* = 3). No significant differences among strains (*p*0.05) indicate comparable cholesterol assimilation capacity under the tested conditions.

Several probiotics have been explored as modern therapies for the management of hypercholesterolemia (Shobharani and Halami, 2016). Multiple mechanisms have been suggested for this attribute by probiotics; in the present study, cholesterol assimilation activity was evaluated by measuring residual cholesterol in the supernatant/cell-free extract (Pereira and Gibson, 2002). Substantial cholesterol assimilation was observed as shown in Figure 5, with values of 64.26 ± 3.0% for ZBOE6, 61.22 ± 5.4% for ZBRV8, and 61.85 ± 6.2% for ZBTM2 (*p* > 0.05), indicating comparable assimilation capacity among the strains. The assimilation is likely to be associated with multiple mechanisms, including BSH-mediated bile acid deconjugation, as well as direct cellular uptake and incorporation into bacterial membranes. In addition, genomic analysis revealed the presence of metabolic genes involved in carbohydrate utilization and cofactor biosynthesis (e.g., riboflavin cluster), which may support cellular processes contributing to cholesterol interaction and assimilation. However, it is important to note that cholesterol assimilation observed under *in vitro* conditions does not directly translate to *in vivo* cholesterol lowering effects. Therefore, these finding should be interpreted as indicative of cholesterol indication potential rather than a confirmed cholesterol lowering effect, and further *in vivo* studies are required to establish their physiological relevance.

#### *In vitro* cytotoxicity studies via the MTT assay

Vero cells, derived from African green monkey kidney epithelium, are widely used as a standard *in vitro* model for preliminary cytotoxicity screening because of their high sensitivity towards toxic compounds and established use in pharmacological safety assessments (Ripert et al., 2016). A cytotoxicity study of the isolates was performed on Vero cell lines at different concentrations (10^6^, 10^7^, and 10^8^ CFU/mL) as well as their respective cell-free extracts.

**Figure 6:**
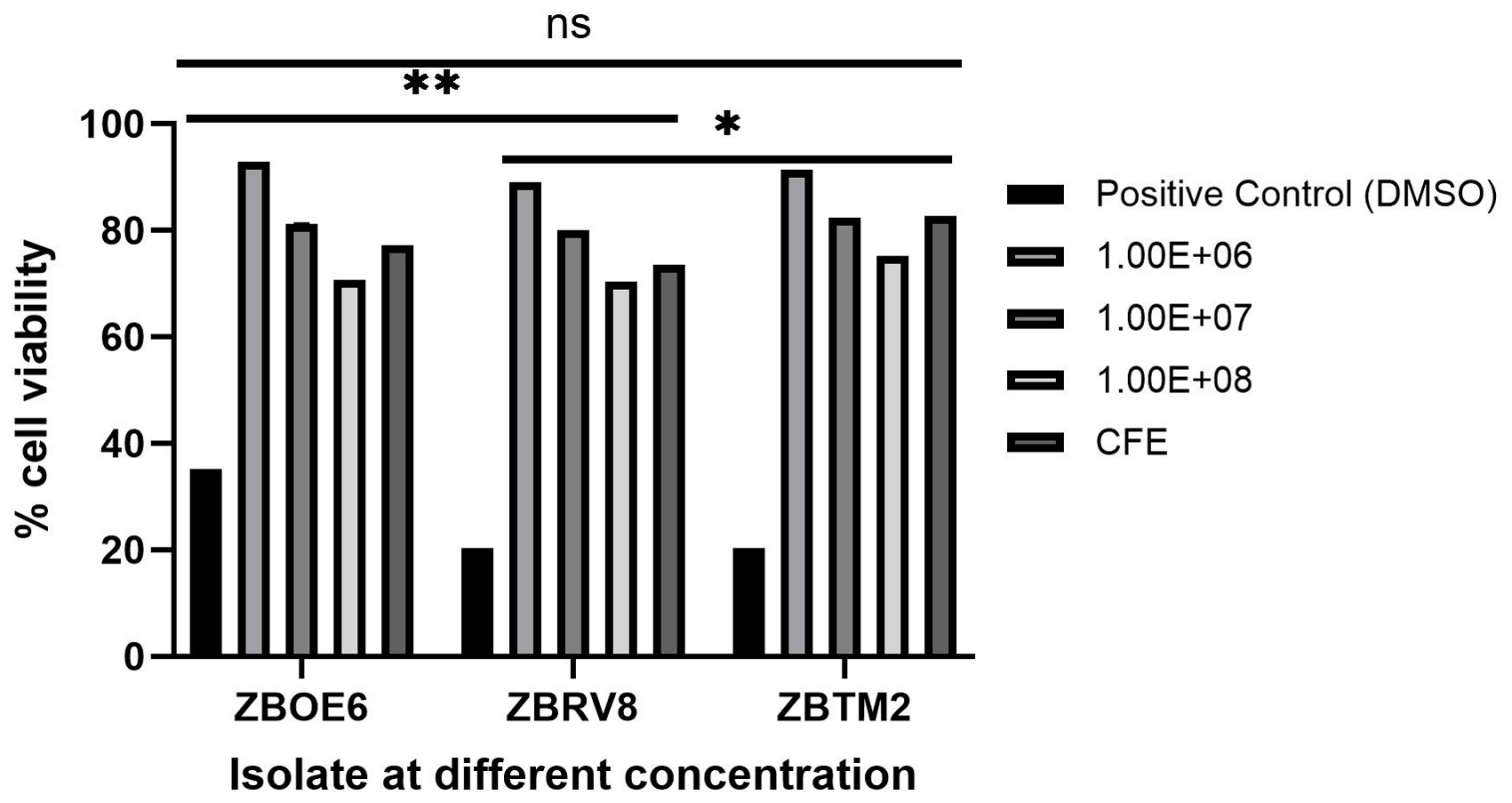
Cytotoxicity assessment of isolates on Vero cell line using the MTT assay. Vero cells were exposed to three different concentrations (1.0 × 10^6^, 1.0 × 10^7^, and 1.0 × 10^8^ CFU/mL) of isolates and their cell-free extracts (CFEs). Cell viability was expressed as a percentage relative to untreated control cells, and the DMSO-treated cells were used as a positive cytotoxic control. Each value represents the mean ± SD (*n* = 3). All the isolates exhibited high viability (> > 70%) across all concentrations and CFEs, indicating low cytotoxic potential. *p*-value ≤ 0.05; ^*^ < 0.05; ^**^ < 0.01; ns, not significant.

High levels of cell viability were observed (Figure 6) ranging from 70.4 to 92.9% even at the highest dose, indicating a low cytotoxicity potential of the isolates. ZBOE6 resulted in the highest viability at 10^6^ CFU/mL (92.89 ± 0.001%), followed by ZBRV8 (91.345 ± 0.003%) and ZBTM2 (89.006 ± 0.005%). The positive control (DMSO) significantly reduced cell viability, confirming the sensitivity of the assay. Similar findings have been reported where *Bacillus subtilis* and *Bacillus coagulans* strains showed negligible cytotoxicity (Poormontaseri et al., 2017; Lefevre et al., 2017).

#### Adhesion assay of the Caco-2 cell line

**Figure 7:**
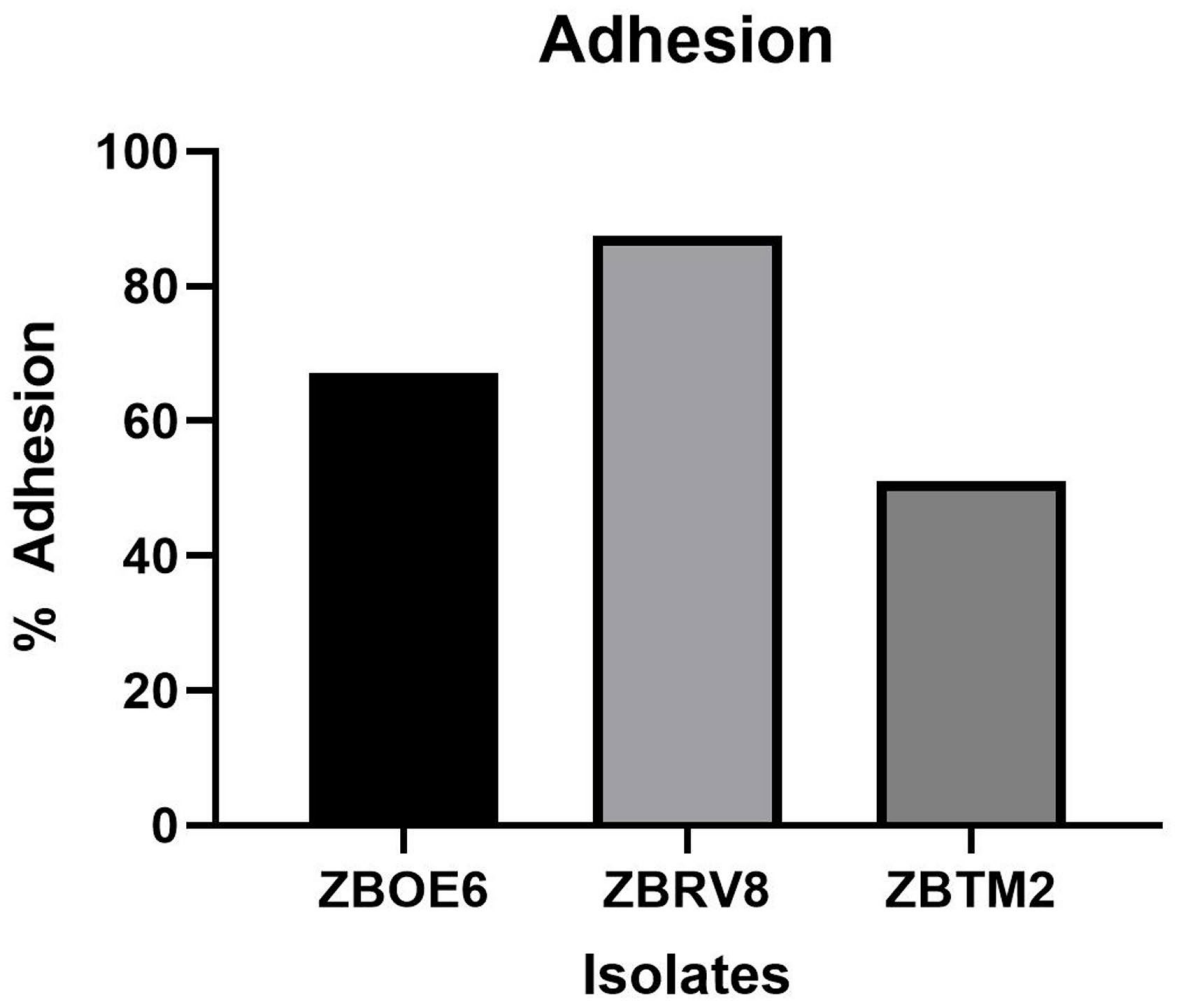
Adhesion ability of isolates ZBOE6, ZBRV8, and ZBTM2 on Caco-2 cell monolayers to assess their potential for epithelial colonization.

Adherence of probiotics to the intestinal mucus layer is a key factor that supports its survival in the gut and contributes to their health promoting effects (Krausova et al., 2019). To simulate the adhesion of probiotics to intestinal epithelial cells, human tumorigenic cell lines, such as Caco-2 and HT-29, have been commonly used (Monteagudo-Mera et al., 2019). ZBOE6, ZBRV8, and ZBTM2 were also evaluated in the human epithelial Caco-2 cell line, and the results are shown in Figure 7.

Among the tested strains, ZBRV8 exhibited the highest adhesion efficiency (87.5%), followed by ZBOE6 (67.0%) and ZBTM2 (51.0%), notably exceeding/similar to typical adhesion values reported for *Bacillus subtilis* probiotics such as strain PY79 (Ostovan et al., 2021) and MBLB2796 (Hwang et al., 2025). These findings of the strains demonstrate good adhesion efficiency, supporting strain-dependent adhesion to intestinal epithelial cell lines, suggesting favourable adhesion properties for intestinal persistence which warrants further *in vivo* validation (Rohith and Halami, 2021; Shinde et al., 2019).

#### Inhibition of pathogenic bacterial adhesion to Caco-2 cells (invasion assay)

The antagonistic potential of the isolates to inhibit the adhesion of *E. coli* to intestinal epithelial cell lines was assessed via competition, exclusion, and displacement assays (Table 5).

In the competition assay, ZBTM2 presented the greatest reduction (58.70%) than ZBOE6 (35.71%) and ZBRV8 (39.13%). The exclusion assay revealed a similar pattern, where pre-colonization by ZBTM2 led to a 58.70% reduction in *E. coli*, compared with 46.74% by ZBRV8 and 36.90% by ZBOE6. Notably, in the displacement assay, ZBTM2 showed the greatest reduction of 78.26%, indicating a strong potential to compete with previously adhered *E. coli*, followed by ZBOE6 (57.14%) and ZBRV8 (42.39%). The ability of these isolates to reduce *E. coli* likely involves production of metabolites inhibiting pathogen adhesion, competition for adhesion site via epithelial binding, biosurfactant production, preventing pathogen attachment, or stimulation of host-defence factors. These *in vitro* data suggest potential anti-adhesive property of the strains against pathogens, warranting mechanistic/*in vivo* confirmations.

#### Resistance to simulated gastric and intestinal conditions

To assess the robustness of the probiotic isolates under gastrointestinal conditions, their percentage survival was evaluated by exposing the spore suspensions of the isolates to artificial gastric (1 h, 2 h) and intestinal (2 h, 4 h) fluids.

**Figure 8:**
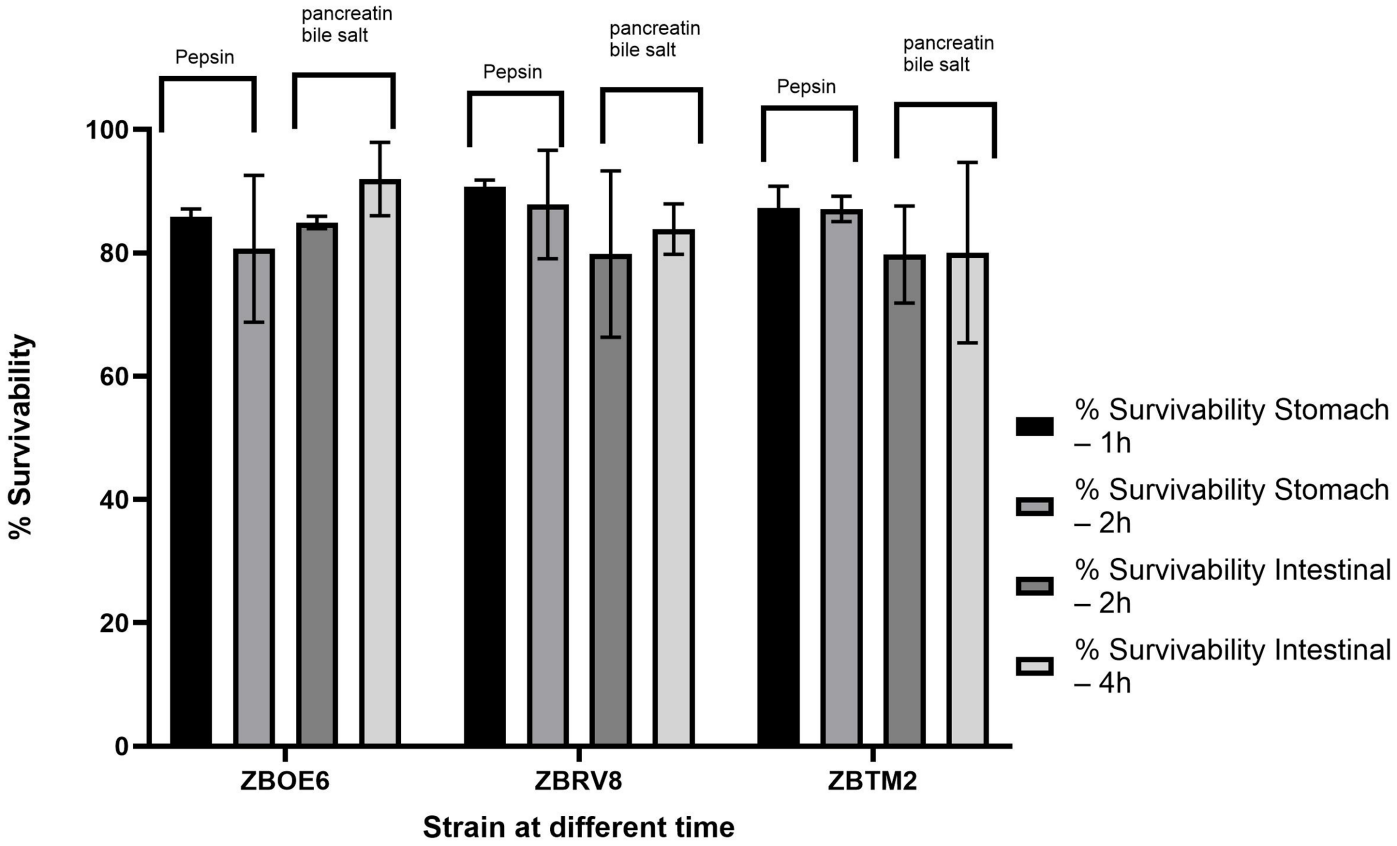
Percentage survivability of isolates under simulated gastrointestinal conditions. Spore suspensions of the isolates were subjected to simulated gastric condition containing 0.3% pepsin at pH 3.0 followed by exposure to simulated intestinal conditions consisting of 0.1% pancreatin with 0.3% bile salts at pH 7.5 for defined time periods. Viability was expressed as the percentage of CFU remaining relative to the initial count (0 h). The results are denoted as mean ± SD (*n* = 3). Statistical analysis using one-way ANOVA followed by Tukey’s post-hoc test indicated no significance differences in survival among the strains (*p* > 0.05).

All the isolates demonstrated good survival across the different time points as shown in Figure 8, ranging from 79.74 to 91.99%. ZBOE6 slightly decreased survival from 85.86% ± 1.29 at 1 h to 80.67% ± 11.92 at 2 h in the gastric simulation but notably increased to 91.99% ± 5.93 at 4 h under intestinal conditions. ZBRV8 maintained high survival throughout the experiment, with 90.75% ± 1.06% survival at 1 h, and the survival rate remained consistent at later time points. ZBTM2 showed the most stable performance, with minimal fluctuations between the gastric and intestinal phases. This indicates that strain can survive the upper digestive tract. Statistical analysis via one-way ANOVA revealed no significant differences among the strains (*p* > 0.05), indicating comparable levels of tolerance across all the tested isolates. Similar findings have been reported where different *Bacillus* strains demonstrated significantly high tolerance to gastric/intestinal conditions (Jlidi et al., 2022).

#### Biogenic amine production capacity

The screening of BA production is a critical safety aspect when developing probiotics for human applications. BA can cause toxic effects in humans, including headaches, hypertension, and allergic reactions, when it is present at high concentrations (Barbieri et al., 2019; Lee et al., 2019). All three isolates (Table 6) exhibited negative reaction to ornithine and histidine, indicating the non-biogenic amine formation (Lee et al., 2017).

**Table 6:** Inhibition of *E. coli* ATCC 8739 adhesions to Caco-2 cells by probiotic isolates through competition, exclusion, and displacement assays. Initial and final CFU/mL values of adhered *E. coli* ATCC® 8739™ was used for the calculation of reduction percentage.

Assay Type	Assay condition	Initial CFU/mL of *E. coli* ATCC® 8,739™	Final CFU/mL of *E. coli* ATCC® 8,739™	Reduction of adhesion in % *E. coli* ATCC® 8,739™
Competition assay	Against ZBOE6	4.20E+07	2.70E+07	35.71
	Against ZBRV8	4.60E+07	2.80E+07	39.13
	Against ZBTM2	4.60E+07	1.90E+07	58.70
Exclusion assay	Against ZBOE6	4.20E+07	2.65E+07	36.90
	Against ZBRV8	4.60E+07	2.45E+07	46.74
	Against ZBTM2	4.60E+07	1.90E+07	58.70
Displacement assay	Against ZBOE6	4.20E+07	1.80E+07	57.14
	Against ZBRV8	4.60E+07	2.65E+07	42.39
	Against ZBTM2	4.60E+07	1.00E+07	78.26

#### Enzyme production

Enzyme production is one of the key functional properties that is enhanced to support digestion, improve nutrient uptake, maintain gut health, and provide broader metabolic benefits (Tshisikhawe et al., 2023). The enzyme production potential of the strains was assessed to understand the functional capabilities (production of dietary enzymes) relevant to probiotic use.

As shown in Table 7, all the isolates lacked protease activity under the tested conditions. Isolate ZBOE6 showed no amylase activity but presented the highest lipase activity (1.58 ± 0.07) and moderate phytase activity (2.88 ± 0.56), although it was negative for *β*-galactosidase. In contrast, isolate ZBRV8 exhibited moderate lipase (1.23 ± 0.12) and phytase (3.02 ± 0.27) activities and was positive for β-galactosidase, indicating potential lactose-digestive support. ZBTM2 uniquely displayed amylase activity (1.22 ± 0.15) along with lipase (1.19 ± 0.05) and phytase (2.67 ± 0.20) and tested positive for β-galactosidase. From a pharmacological perspective, phytase activity is a valuable trait with respect to the hydrolysis of dietary phytates, which are abundant in cereals, legumes, nuts, and seeds. Phytase chelates essential minerals such as iron, calcium, and zinc, thereby increasing their intestinal bioavailability (Selle and Ravindran, 2008). Lipase production can complement the production of pancreatic enzymes, facilitating the efficient digestion and absorption of dietary fats (Drouault et al., 2002). Lipase activity also helps in lowering the triglycerides by metabolizing into fatty acids for its easy assimilation. Furthermore, β-galactosidase positive strains such as ZBRV8 and ZBTM2 can hydrolyse lactose into glucose and galactose, suggesting potential to support lactose digestion. This property of the strains may help in lactose intolerance symptoms and improving dairy tolerance in susceptible individuals which can be further validated through *in vivo* studies (Oak and Jha, 2019). Collectively, these enzyme-associated traits strengthen the role of these isolates in digestion and metabolism, extending their probiotic potential (Table 8).

**Table 7:** Biogenic amine formation of the isolates in broth.

*Isolate*	Absorbance	pH	Colour
ZBOE6-Control	0.11 ± 0.01	5.03 ± 0.05	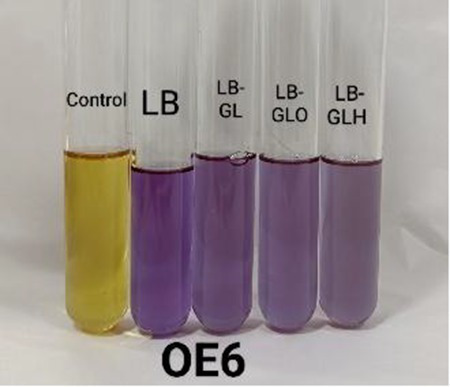
ZBOE6-LB	0.99 ± 0.03	7.58 ± 0.38	
ZBOE6-LB-GL	0.78 ± 0.03	6.48 ± 0.55	
ZBOE6-LB-GLO	0.69 ± 0.06	6.23 ± 0.79	
ZBOE6-LB-GLH	0.64 ± 0.07	6.18 ± 0.60	
ZBRV8-Control	0.11 ± 0.00	5.01 ± 0.03	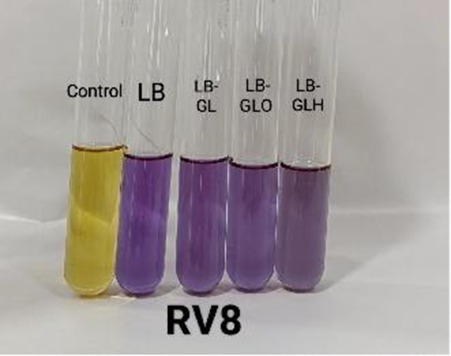
ZBRV8-LB	0.99 ± 0.00	7.55 ± 0.36	
ZBRV8-LB-GL	0.75 ± 0.15	6.69 ± 0.35	
ZBRV8-LB-GLO	0.64 ± 0.16	6.18 ± 0.80	
ZBRV8-LB-GLH	0.62 ± 0.13	5.88 ± 0.67	
ZBTM2-Control	0.13 ± 0.04	5.06 ± 0.21	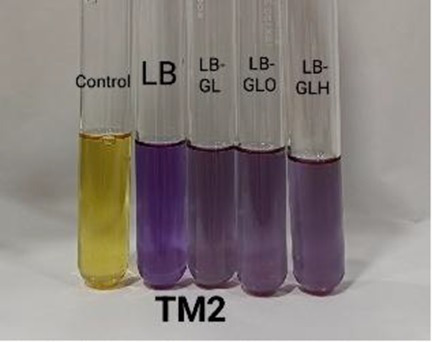
ZBTM2-LB	0.98 ± 0.03	7.35 ± 0.09	
ZBTM2-LB-GL	0.64 ± 0.01	6.36 ± 0.18	
ZBTM2-LB-GLO	0.55 ± 0.03	5.98 ± 0.37	
ZBTM2-LB-GLH	0.56 ± 0.03	5.80 ± 0.57	

The results are expressed as the mean ± SEM (*n* = 3). LB: Luria–Bertani broth; GL: LB supplemented with glycerol (0.25%); GLO: LB supplemented with glycerol (0.25%) and ornithine (0.1%); GLH: LB supplemented with glycerol (0.25%) and histidine (0.1%).

**Table 8:** Enzymatic profile of isolates based on substrate hydrolysis and ONPG assays.

Isolate	Amylase	Protease	Lipase	Phytase	β-galactosidase
ZBOE6	nd	nd	1.58 ± 0.07	2.88 ± 0.56	Negative
ZBRV8	nd	nd	1.23 ± 0.12	3.02 ± 0.27	Positive
ZBTM2	1.22 ± 0.15	nd	1.19 ± 0.05	2.67 ± 0.20	Positive

The results are expressed as mean ± SEM (*n* = 3). Values represent zone of inhibition diameter (mm) for hydrolysis assays. Nd, not detected, positive/negative for β-galactosidase activity based on colour development in ONPG broth.

#### Antioxidant activity

**Figure 9:**
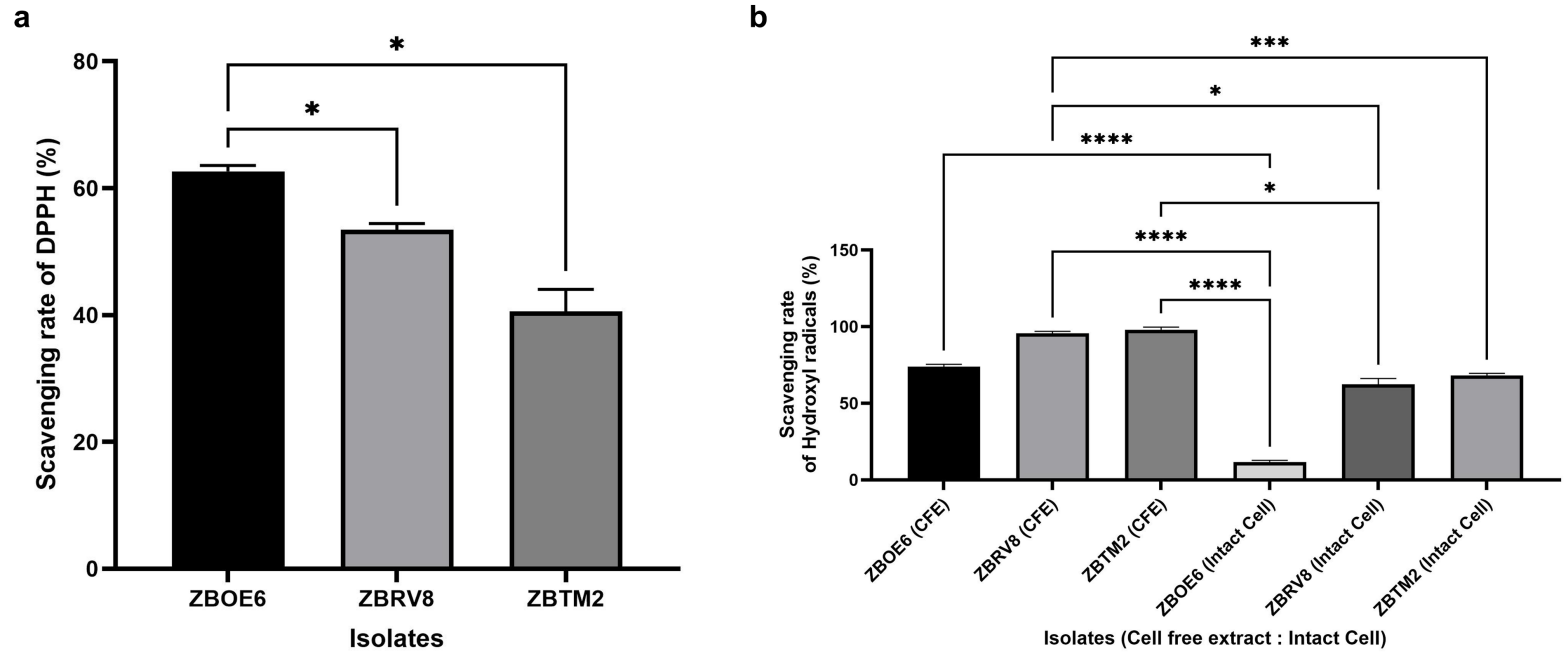
Antioxidant activity of selected isolates measured via DPPH **(a)** and hydroxyl radical scavenging assays **(b)**. Data represent mean ± SD (*n* = 3). DPPH activity was measured using intact cells whereas intact cells and cell-free extracts were tested for hydroxyl radical scavenging activity. Statistical significance (*p* < 0.05) was denoted using two-way ANOVA with Tukey’s post-hoc test. Statistical significances are denoted as ^*^p < 0.05, ^***^p < 0.001, and ^****^p < 0.0001.

Oxidative agents, including reactive oxygen species (ROS) and reactive nitrogen species (RNS), can produce free radicals that can lead to extensive oxidative damage to cellular lipids, membranes, proteins, and DNA (Mazhar et al., 2024; Simon et al., 2023). Isolates were evaluated for their ability to scavenge DPPH radicals and hydroxyl radicals. All three isolates exhibited distinct patterns as illustrated in Figure 9.

In the DPPH assay (Figure 9a), ZBOE6 had the highest scavenging capacity (62.67 ± 0.91%), followed by ZBRV8 (53.45 ± 1.09%) and ZBTM2 (40.60 ± 2.00%), with significant differences between isolates (*p* < 0.05). Similar findings have been reported for other *Bacillus* strains (Khan et al., 2023). The reported DPPH % activity for each strain reflects their capacity to neutralize free radicals compared with the known antioxidant standard ascorbic acid. For hydroxyl radical scavenging (Figure 9b), ZBTM2 demonstrated the highest activity in both forms, with 97.93 ± 1.72% for intact cells and 68.09 ± 1.53% for CFE. ZBRV8 also showed strong antioxidant potential with 95.56 ± 1.33% (intact) and 62.45 ± 3.80% (CFE). In contrast, ZBOE6 exhibited moderate activity in its intact form (73.84 ± 1.55%) and significantly lower CFE-based scavenging (11.70 ± 1.10%). These results align with previous reports suggesting strain-specific differences in antioxidant capacity due to the cellular localization of active biomolecules (Chen et al., 2014).

## Discussion

This study represents the isolation and comprehensive *in vitro* characterization of three *Bacillus subtilis* strains (ZBOE6, ZBRV8, and ZBTM2) isolated from ethically sourced neonatal meconium, an unexplored microbial niche. These isolates demonstrated multiple *in vitro* traits associated with probiotic potential, supported by whole-genome sequencing-based characterization. *In vitro* assays indicated promising probiotic-associated attributes supported by whole-genome sequencing. The isolates exhibited non-haemolytic (α/γ-type) nature, DNase negativity, and absence of biogenic amine production, supporting their preliminary safety profile.

Gastrointestinal resilience was suggested by acid tolerance (78–95% survival at pH 2.0), bile tolerance (65–89% at 0.3% bile), and simulated GI transit survival (>79%). These phenotypes are mechanistically supported by the presence of stress response regulators (*perR*, *sigB*, and *fur*), oxidative stress enzymes (*soda/C and kat*), and osmotic adaptation system (*opu* operon), which collectively contribute to the cellular homeostasis under gastrointestinal stress conditions. Notably, the absence of *sigB* in ZBRV8 may partially explain its relatively lower stress tolerance than ZBOE6 and ZBTM2, indicating strain level functional differentiation. BSH activity and cholesterol assimilation (~60–64%) suggest a potential role in bile metabolism and cholesterol interaction; however, these effects are likely multifactorial, involving BSH-mediated bile deconjugation as well as possible membrane incorporation or co-precipitation mechanisms. Importantly, these observations are derived from *in vitro* conditions and should not be directly interpreted as evidence of *in vivo* validation cholesterol lowering efficacy (Shobharani and Halami, 2016).

Adhesion properties of the strains appear promising with Caco-2 attachment up to 87.5%, high surface hydrophobicity (71–83% with hexane), and good auto/co-aggregation. These traits may facilitate epithelial interaction and competitive exclusion of pathogens. Genomic identification of biofilm-associated loci *(tasA, sinR, yqxM, and sinR/I)* provides a mechanistic basis of these observations; however, variation in regulatory components (absence of *sinl* in ZBRV8) may contribute to the observed strain-specific differences in adhesion efficiency. This highlights genotype–phenotype relationship rather than a simple presence-based interpretation.

Safety profiling confirmed non-haemolytic/DNase negative phenotype, no biogenic amines (Krausova et al., 2019; Barbieri et al., 2019), low cytotoxicity on the Vero cell line (AlGburi et al., 2016), and EFSA compliant antibiotic susceptibility (EFSA Panel on Additives and Products or Substances used in Animal Feed (FEEDAP), 2012; Shin et al., 2021). Phenotypic testing revealed tetracycline resistance in ZBOE6 (32 μg/mL), and streptomycin (21 μg/mL) and clindamycin (1.8 μg/mL) resistance in ZBRV8, with MICs exceeding EFSA thresholds for *Bacillus* sp. These findings indicate that the observed resistance patterns are consistent with the intrinsic characteristic of the *B. subtilis*, such as chromosomally encoded efflux systems (e.g., *bcrA/C*) and ribosomal protection mechanism, rather than acquired resistance determinants. Whole-genome analysis using CARD and VFDB further confirmed the absence of transferable antimicrobial resistance genes (threshold >90% identity and 60% coverage) as well as lack of mobile genetic elements. Importantly, these results align with Rychen et al. (2018) criteria, which distinguish intrinsic, non-transmissible resistance from acquired resistance of concern. Notably, strain ZBTM2 exhibited complete susceptibility to all tested antibiotics. Overall, the antibiotic resistance profile comply with the Qualified Presumption of Safety framework supporting the safety of these strains for probiotic applications (EFSA Panel on Biological Hazards (BIOHAZ) et al., 2026).

Multienzyme activity including lipase, phytase, amylase, and β-galactosidase together with notable antioxidant capacity suggests functional versatility of the strains. The potential is further supported by the presence of key metabolic genes, such as *bglA*, and the riboflavin biosynthetic gene cluster which are associated with carbohydrate metabolism and antioxidant production, respectively (Tshisikhawe et al., 2023).

A key limitation of this study is its *in vitro* scope; therefore, *in vivo* and clinical investigations are necessary to validate the observed effects of gut persistence, pathogen exclusion, cholesterol modulation, and related health benefits. Nonetheless, these strains demonstrate strong potential as functional candidates for food applications targeting gut and metabolic health, subject to further *in vivo* validation.

## Conclusion

This study presents a comprehensive *in vitro* characterization of *Bacillus subtilis* strains isolated from neonatal meconium, an unexplored niche that may harbour strains with probiotic potential. These selected strains (ZBOE6, ZBRV8, and ZBTM2) demonstrated a combination of favourable safety attributes, stress tolerance, adhesion capacity, and functional properties, including enzyme production, antioxidant activity, and cholesterol assimilation. Integration of phenotypic assays with genome-based analysis provided preliminary mechanistic insight into strain-specific functional traits, particularly in relation to stress response, adhesion, and metabolic potential. While the results suggest that these isolates are promising probiotic candidates, it is important to emphasize that all the findings are derived from *in vitro* and *in silico* analyses. Therefore, their functional efficacy, safety, and stability must be validated through *in vivo* studies and clinical trials before any application on functional food or therapeutic interventions. Taken together, this study provides a foundation of further investigation of these strains, highlighting their potential while acknowledging the need of comprehensive *in vivo* validations.

## Data Availability

The datasets presented in this study can be found in online repositories. The names of the repository/repositories and accession number(s) can be found in the article/supplementary material.
